# Comparison of Antibacterial Mode of Action of Silver Ions and Silver Nanoformulations With Different Physico-Chemical Properties: Experimental and Computational Studies

**DOI:** 10.3389/fmicb.2021.659614

**Published:** 2021-07-01

**Authors:** Anna Kędziora, Robert Wieczorek, Mateusz Speruda, Iva Matolínová, Tomasz M. Goszczyński, Ireneusz Litwin, Vladimír Matolín, Gabriela Bugla-Płoskońska

**Affiliations:** ^1^Department of Microbiology, Faculty of Biological Sciences, University of Wrocław, Wrocław, Poland; ^2^Faculty of Chemistry, University of Wrocław, Wrocław, Poland; ^3^Department of Surface and Plasma Science, Faculty of Mathematics and Physics, Charles University, Prague, Czechia; ^4^Laboratory of Biomedical Chemistry, Department of Experimental Oncology, Hirszfeld Institute of Immunology and Experimental Therapy, PAS, Wrocław, Poland; ^5^Department of Genetics and Cell Physiology, Faculty of Biological Sciences, University of Wrocław, Wrocław, Poland

**Keywords:** *Escherichia coli*, gram-negative bacteria, silver, nanoformulations, mode of action, computational method

## Abstract

The aim of this study was to compare the antibacterial mode of action of silver ions (Ag^+^) and selected silver nanoformulations against *E. coli* strains (*E. coli* J53, *Escherichia coli* BW25113 and its derivatives: Δ *omp*A, Δ *omp*C, Δ *omp*F, Δ *omp*R, ompRG596AcusSG1130A, cusSG1130A). In this research we used various experimental methods and techniques such as determination of the minimal inhibitory concentration, flow cytometry, scanning electron microscopy, circular dichroism as well as computational methods of theoretical chemistry. Thanks to the processing of bacteria and silver samples (ions and nanoformulations), we were able to determine the bacterial sensitivity to silver samples, detect reactive oxygen species (ROS) in the bacterial cells, visualize the interaction of silver samples with the bacterial cells, and identify their interactions with proteins. Differences between the mode of action of silver ions and nanoformulations and the action of nanoformulations themselves were revealed. Based on the results of computational methods, we proposed an explanation of the differences in silver-outer protein interaction between silver ions and metallic silver; in general, the Ag^0^ complexes exhibit weaker interaction than Ag^+^ ones. Moreover, we identified two gutter-like areas of the inner layer of the ion channel: one more effective, with oxygen-rich side chains; and another one less effective, with nitrogen-rich side chains.

## Introduction

Ionic silver has been known for its antibacterial properties for centuries, but fast development of nanotechnologies has allowed the use of silver in the form of silver nanoformulations (SNF). For the last years, SNF have been one of the most popular antibacterial agents, and we can observe their extensive use in and outside of the clinic. The SNF variety of available forms is huge. Depending on the synthesis method and purpose, they can be directly present in the form of a colloid, suspension, powder, gel or incorporated into intermediate products such as cosmetics (e.g., intended for acne-prone skin), building materials (e.g., paints, varnish), textiles (e.g., underwear, clothes, bedding), household items (e.g., washing machines, refrigerators), electronic devices (e.g., telephones, keyboards), cleaning products (e.g., cleaning fluids), supplements, dressings, and ointments. They differ from each other in physico-chemical properties, such as size, shape, charge, surface area, oxidation state, compounds, and bioavailability (Kędziora et al., 2018; Pareek et al., 2018; Zheng et al., 2018). It should be underlined that differences between the physico-chemical properties of SNF result in potentially different antibacterial modes of action and various bacterial responses to them (Pareek et al., 2018; Zheng et al., 2018; Kędziora et al., 2020). The mode of the antibacterial activity of silver ions and the mechanism of bacterial resistance to them have been well described in the literature (Pareek et al., 2018; Bonilla-Gameros et al., 2020). There are also many attempts to explain how SNF work, but the results can be conflicting (Li et al., 1997; Banerjee et al., 2010; Liu et al., 2012; Radzig et al., 2013; Randall et al., 2015; Borkowski et al., 2016; Reymond-Laruinaz et al., 2016; Zhang et al., 2018; Zheng et al., 2018; Anuj et al., 2019; Siddiq et al., 2019; Wu et al., 2019). SNF can interact with the bacterial membrane, cell wall or subcellular structure, causing their damage or function disruption (through generations of reactive oxygen species (ROS) or affinity toward intracellular structure) in different ways than silver ions, and differences between individual SNF forms might also be observed (Pareek et al., 2018; Zheng et al., 2018; Kędziora et al., 2020). In our previous work, we determined the consequences of long-term exposure of bacteria to SNF with different physico-chemical properties (Kędziora et al., 2020). In the case of some forms of nanosilver, 90% of the bacterial population was able to change its sensitivity not only to silver itself, but also to antibiotics, while in the case of others, this sensitivity was not disturbed. Initial analysis showed that it may be the cause of genetic (mutational) or phenotypical (adaptive) changes depending on the applied form of SNF and pathways of silver uptake to bacterial cells (Kędziora et al., 2020).

Some outer membrane proteins, such as OmpC and OmpF, are arranged in the uptake of silver ions into the cell (Li et al., 1997; Liu et al., 2012; Radzig et al., 2013; Randall et al., 2015), and might also be responsible for some kinds of silver nanoparticles entering (Li et al., 1997; Radzig et al., 2013). Randall et al. (2015) reported that point mutations in the *omp*R genes encoding the regulatory proteins OmpR for *omp*C and *omp*F expression cause high resistance to silver ions. In this way, they identified endogenous resistance of bacteria to silver ions – another form, apart from the known and documented one, of exogenous resistance (horizontally acquired in Gram-negative bacteria) encoded on the plasmid pMG101 as the *sil* operon. *Sil* genes encode structural proteins located in the outer membrane and periplasm (SilA, SilB, SilC, SilE, SilF, SilP), and regulatory proteins (SilP, SilR) (Randall et al., 2015). Moreover, in bacteria with endogenous silver resistance CusCFBA transporter (regulated with CusR after sensor kinase – CusS – phosphorylation) was found to be responsible for active silver efflux (Randall et al., 2015).

The scientific justification of the topic is related to the explanation of the risk of bacterial resistance to nanosilver and other biocides and is an important aspect for the global public health. For a deeper explanation of our previous observations, suspicions and literature data, we decided to compare the silver ions’ and selected nanoformulations’ mode of action via experimental and computational methods. To investigate the differences in the interaction of Ag^+^ and SNF with bacterial cells we performed experiments with strains with a typical and altered cell structure (*Escherichia coli* BW25113 wt and derivates with certain OMP outer membrane proteins such as OmpA, OmpC, OmpF, OmpR and/or CusS) (Randall et al., 2015). Moreover, we used the silver-ions resistant strain of *E. coli* J53 (genetic determinant in the presence of plasmid pMG101). Determination the sensitivity of tested bacteria strains to applied silver materials (ions and SNF) showed us the participation of selected outer membrane proteins in silver uptake and their efficacy. The level of reactive oxygen species (ROS) and the time of their appearance in bacteria cells after silver treatment reflected the cellular response to different physico-chemical properties of silver and outer membrane structure. The differences in the interaction of silver ions and nanomaterials with model bacteria cell *E. coli* BW25113 wt were also visualized with field emission scanning electron microscopy (FE-SEM). Human serum albumin, a standard protein used in circular dichroism (CD), due to its high purification, provided us with information on the interaction of silver ions and nanoformulations with the model protein. Computational methods of theoretical chemistry were used as a useful tool to predict the structure and stability of silver ions and nanomaterials with a certain outer membrane protein (OMP) located in a bacterial cell and involved in silver uptake (we chose OmpF nd OmpC) (Wieczorek et al., 1999; Mielke et al., 2000; Salvador et al., 2007; Rudowska et al., 2013; Olszewski, 2015).

## Materials

### Bacteria Strain

*E. coli* J53, *Escherichia coli* BW25113 and its derivatives (Δ *omp*A, Δ *omp*C, Δ *omp*F, Δ *omp*R, ompRG596AcusSG1130A and cusSG1130A) were tested. Usage of the bacteria strains deprived of some major outer membrane porins gave as possibilities to determine the participation of outer membrane proteins in SNF uptake and the differences between SNF mode of action. All tested strains are described in details in Table 1. Strains were provided by a team from University of Leeds.

**TABLE 1:** Detailed description of bacterial strains.

		Description	References
Strains	*E. coli* BW25113 (wt)	Wild type, complete Omp	Randall et al., 2015
	*E. coli* BW25113 Δ *omp*C	Deleted OmpC from the OM	Randall et al., 2015
	*E. coli* BW25113 Δ *omp*F	Deleted OmpF from the OM	Randall et al., 2015
	*E. coli* BW25113 Δ *omp*R	Deleted OmpR from the cell	Randall et al., 2015
	*E. coli* BW25113 Δ *omp*A	Deleted OmpA from the OM	Randall et al., 2015
	*E. coli* BW 25113ompRG596AcusSG1130A (AgR)	Point mutation in the *omp*R and *cusS* after silver ions exposure, finally lack of OmpC, OmpF in the OM and CusS inside the cell	Randall et al., 2015
	*E. coli* BW25113 cusSG1130A	Point mutation in the *cusS* after silver ions exposure, finally lack of CusS in the bacteria cell	Randall et al., 2015
	*E. coli* J53	Reference strains resistant to silver ions due to possessing the plasmid pMG101 in the cell related with Sil proteins in the OM	Randall et al., 2015; Kędziora et al., 2020

### Silver Samples

The silver samples used in this study were chosen based on our previous results (Kędziora et al., 2020) and they are as follows: silver ions (Ag^+^) as inner control, silver nanoparticles immobilized on amorphous TiO_2_ (TiO_2_/Ag^0^) with a silver size of 20 nm (marked as S2), and an aqueous dispersion of silver nanoparticles stabilized with a trace amount of Tween and polyethyleneimine with a silver size of 20 nm (marked as S7) (Kędziora et al., 2020). Selected S2 and S7 exerted a selective pressure to some bacteria strains. In Gram-negative bacteria (*Escherichia coli*, *Klebsiella pneumoniae*, *Enterobacter aerogenes*), after their exposure to samples S2 and S7, we observed an increase in bacterial resistance to S2 and S7 samples due to huge genetic changes such as point mutations in the case of S2, and single genetic changes and probably wide phenotypic changes after the action of sample S7 and their sensitivity to antibiotics, including increasing bacterial susceptibility to some classes of antibiotics. They differ from each other in size, shape, surface area, compounds, and bioavailability. Silver ions (Ag^+^, AgNO_3_), nanosilver embedded in TiO_2_ (marked as S2) and water dispersed silver nanoparticles (marked as S7) were used as tested samples. All details are described in our previous study (Kędziora et al., 2020).

## Methods

### Sensitivity of *E. coli* Strains (*E. coli* J53, *E. coli* BW25113 and Its Derivatives) to Silver Samples

The sensitivity of bacterial strains to silver samples was determined by Clinical and Laboratory Standards Institute (CLSI) methods (Weinstein, 2006). Muller Hinton Broth (MHB) and Muller Hinton Agar (MHA) were used as medium. Bacterial strains were stored at −70°C and prior to each investigation the standard protocol to check their purity and revitalization was performed. The inoculum was spread on an agar plate (MHA), incubate at 37°C for 19 h and then used for the experiment. Derivatives (*E. coli* BW25113 Δ *omp*A, 25113 Δ *omp*C, Δ *omp*F and Δ *omp*R) were selected using MHA supplemented with kanamycin at 50 mg/L to be sure the purity of derivatives before starting the experiment. During experiment a stock of silver samples (at concentration from 2048 to 0.125 μg/mL) in MHB with a final volume 90 μL was prepared in micro titration plates. Next, the preincubated bacteria strains were established at 0.5 McFarland and added at a final concentration of 1.5 × 10^6^ cfu/mL to each silver sample concentration. Final volume of each well was 100 μL. Controls (pure medium, medium with silver samples stock and medium with bacteria strains) were established. Minimal inhibitory concentration (MIC) values were read within 16–19 h. MIC was estimated by optical measurement and means the bacteriostatic concentration activity of tested samples (no growth was observed). The bactericidal and bacteriolytic effect don’t have to be observed in this case. To verify this assay and determine minimal bactericidal concentration (MBC), which means that more than 99.9% of cell were killed, bacteria were inoculated in agar plates (at volume 10 μL) and the number of colonies were counted. Obtained data of MBC were not added to the manuscript, but they confirmed the obtained results. The experiment was repeated 3 times.

### ROS Detection in Bacterial Cells Using Flow Cytometry

To estimate the levels of intracellular ROS, two fluorescent markers for the total ROS production were used: 2’,7’-dichlorofluorescein (DCFH-DA) and dihydroethidium (DHE), produced by Thermo Fisher Scientific. In the case of 2’,7’-dichlorofluorescein, after crossing the cell membrane DCFH-DA becomes deacetylated by esterases and becomes converted to 2’,7’-dichlorodihydrofluorescein (DCFH_2_). In the presence of a wide range of ROS, DCFH_2_ becomes oxidized to 2’,7’-dichlorofluorescein (DCF) that emits green fluorescence (515–530 nm) after excitation by blue light (∼485–500 nm). The other ROS probe, DHE, passively enters the cell and becomes oxidized by different kinds of ROS. This results in formation of ethidium and 2-hydroxyethidium, both of which can be excited by blue light (∼500 nm), leading to the emission of fluorescence at ∼606 nm (Kar et al., 2014; Borkowski et al., 2016; Zhang et al., 2018; Wu et al., 2019).

The protocol for sample preparation was based on the method described by Banerjee et al. (2010) with our own modifications necessary for the experiment to be carried out on bacteria. After overnight incubation (37°C) of *E. coli* BW25113 wt, *E. coli* BW25113 AgR and *E. coli* J53 the bacterial culture was resuspended in Luria Broth (LB) medium and then incubated for another 4 h. The number of bacterial cells in the suspension was estimated using the McFarland scale. For the experiment, the culture of tested *E. coli* with 0.5 McFarland turbidity was diluted 100-fold to 10^6^ cfu/mL, and then poured into Eppendorf tubes. Each was incubated with 3.75 μL of DCFH-DA or 2 μL of DHE for 15 min (at a final concentration of 5 μM). This time was sufficient for fluorophores to passively penetrate through bacterial cell membranes into the cytoplasm. After incubation, the samples were centrifuged (7,000 rpm; 1.5 min, 20°C) and supernatant with possible overflow of DCFH-DA (or DHE) was discarded. The bacterial pellet was washed twice with deionized water using centrifugation (7,000 rpm; 1.5 min, 20°C). The removal of supernatant containing culture medium was crucial for the protocol – some of the tested compounds may react with components of the LB broth and form a sediment affecting later measurements, so for the next step, the samples were resuspended in Milli-Q water. After the addition of tested silver nanomaterials at MIC (or H_2_O_2_ at a final concentration of 3 mM and menadione at a final concentration 100 μM; as in the case of the control), samples were incubated for 90 min at 37°C. Measurements of generated ROS using flow cytometry were taken every 15 min. The experiment and later analysis of the results were carried out on a Guava easyCyte Flow Cytometer (Merck) using the InCyte application program.

It should be noted that one of the tested compound (S7) was able to emit fluorescence after excitation with laser light. This situation occurred only when there was a high concentration of silver in the samples. Concentration of tested samples did not affect the final results of this research, because it was low enough to be detected by a flow cytometer.

### FE-SEM Observation

The bacterial strain (*E. coli* BW25113) was incubated with silver nanoformulations for 90 min at 37°C. Applied density of bacteria and silver concentration were adjusted to 1 × MIC as previously determined (Kędziora et al., 2020). The treated and control bacterial samples were taken and centrifuged at 10,000 rpm for 2 min followed by phosphate-buffered saline (PBS) washing. This step was repeated twice. Then bacterial samples were drop casted on a silicon wafer surface, dried in the air and observed using a field-emission scanning electron microscope (FE-SEM, MIRA III, Tescan, s.r.o) equipped with an energy dispersive X-ray spectroscopy detector (EDX, XFlash, Bruker) at a primary electron energy of 30 keV.

### Circular Dichroism

To determine the differences in interaction between Ag^+^ and SNF with proteins albumin from human serum (HSA, fatty acid free) was used as a model protein. Circular dichroism (CD) spectra were recorded at 25°C on a J-1500 spectropolarimeter (Jasco, Japan) equipped with a thermostated cell holder. HSA was purchased from Sigma-Aldrich, sodium bicarbonate, analytical grade, was obtained from Avantor Performance Materials (Poland). All solutions were prepared in Milli-Q water (18.2 M cm^–1^) produced by a Direct-Q3 UV system (Millipore, United States). The HSA concentration was 1.5 μM and its concentration was determined by measurement of the absorbance at λ = 278 nm using ε = 44300 M^–1^ cm^–166^. The stock solutions of S2 and S7 were prepared by dilutions original samples to final concentrations of 480 μM and 4 ppm respectively, using NaHCO_3_ (10 mM, pH 8.3). The stock solution of HSA in NaHCO_3_ (10 mM) was 3 μM. The samples for CD measurement were prepared as follows: the stock solution of S2 or S7 was added to the stock solution of HSA in the 1:1 volume ratio and measurements were taken immediately after mixing. Three spectra (recorded with a data pitch of 0.2 nm, band width of 2 nm, and data integration time of 1 s at 100 nm min^–1^) were averaged for each sample. Each measurement was subtracted by NaHCO_3_ (10 mM) supplemented with a suitable amount of S2 or S7. Therefore, the resulting spectrum corresponds to the contribution of HSA alone. CD signals were converted to mean residue molar ellipticities using an average residue weight of 114.2. The fractional contents of α-helices were calculated from CD spectra using the Dichroweb platform (CDSSTR with dataset SP175). This method was impossible to carry out in the case of S2, due to physico-chemical properties of this sample.

### Computational Methods

Molecular orbital studies on the Ag^0^ and Ag^+^ cation 1:1 complex with OmpF and OmpC protein models and solvent (water) model introduced upon potential energy surface investigation were performed using the density functional theory (DFT) with the IEF-PCM (integral equation formalism polarizable continuum model) (Cammi, 2013). The above proteins (OmpF and OmpC) belong to the bacterial proteins of the outer membrane and were selected for this assay based on our previous study (Kędziora et al., 2020). According to the literature and our observation (Kędziora et al., 2020), it is known that these proteins could be involved in the absorption of silver. Information on the molecular structure of OmpF and OmpC was taken from the UNIPROT database (P02931 and P06996). The starting structure of the side chains of the proteins for DFT calculations was generated on the basis of 35 ps simulation at 300 K, without cutoffs using BIO+ implementation of the CHARMM force field. DFT calculations were performed with the Gaussian 09 C.01 (Frisch et al., 2009) suite of programs using the ωB97X-D (Frisch et al., 2009) long-range corrected hybrid density functional with damped atom-atom dispersion corrections. The Stuttgart/Dresden effective core potential (ECP) for Ag and full double-**ζ** D95 basis set for the rest of the atoms were used. All presented structures were fully optimized.

## Results

### Sensitivity of *E. coli* Strains (*E. coli* J53, *E. coli* BW25113 and Its Derivatives) to Silver Samples

**FIGURE 1:**
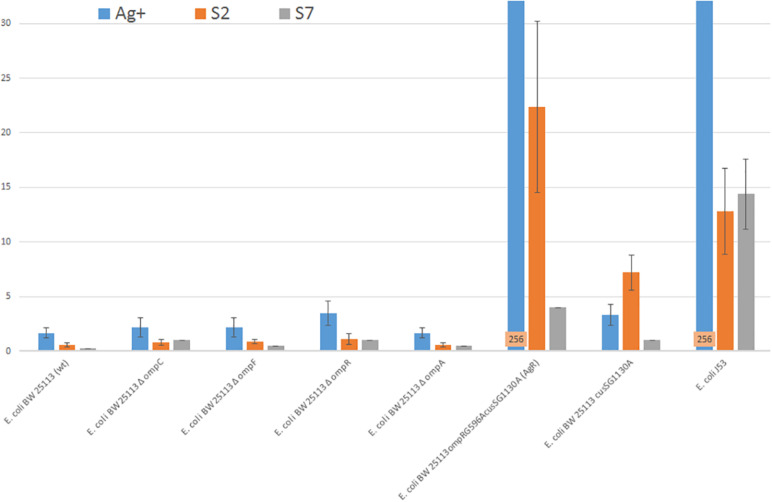
Silver sample’s MIC values of *E. coli* strains: *E. coli* J53, *E. coli* BW25113 (wt) and its derivatives.

The differences between the antibacterial activity of silver ions and silver nanoformulations were observed and are summarized in Figure 1. The differences of sensitivity bacteria lacking single proteins OmpC, OmpF, OmpR, OmpA or CusS to S2, S7 and Ag^+^ are not statistically important. *E. coli* BW25113cusSG1130A deprived CusS protein is the least sensitive to the S2 sample. A comparison of the MICs for *E. coli* BW25113cusSG1130A and *E. coli* BW25113ompRG596AcusSG1130A (AgR), in which, apart from CusS, the OmpR, C and F proteins are missing, allows us to conclude that the OMP proteins (not one, but probably several) are most involved in the transport of Ag^+^ and S2 into the cell. However, the *E. coli* BW25113ompRG596AcusSG1130A (AgR) strain, Ag^+^ resistant, moderately resistant to S2, still remains susceptible to the S7 assay.

### Level of ROS Generated in Bacterial Cells After Treatment With Tested Silver Materials

**FIGURE 2:**
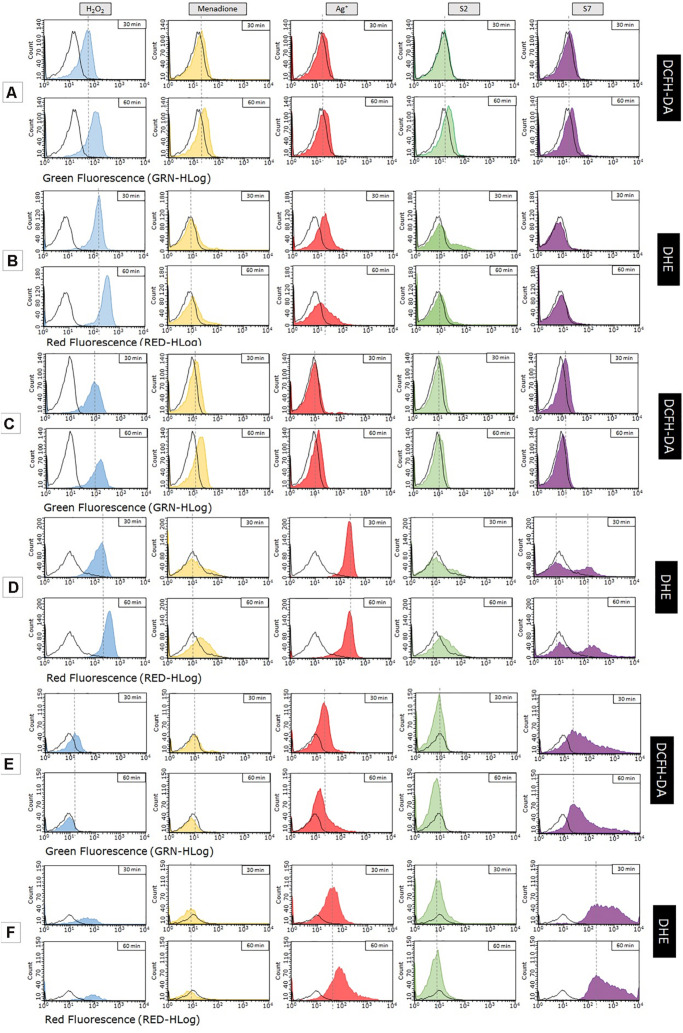
Measurement of reactive oxygen species generated in bacterial cells after exposure to nanosilver compounds with different physico-chemical properties. Research was carried out on 2 fluorescent dyes (DCFH-DA, DHE). Tested strains of *Escherichia coli* – BW25113 **(A,B)**, its mutant marked as AgR **(C,D)** and *E. coli* J53 **(E,F)** – were exposed to MIC concentration of AgNO_3_ (red), S2 (green), S7 (violet), H_2_O_2_ (blue – control), menadione (yellow – control) or left untreated (black – control).

Histograms presented in Figure 2 show the level of ROS generated in *E. coli* BW25113 wt, *E. coli* BW25113 AgR and *E. coli* J 53 strains after exposure to tested Ag^+^ and SNF S2 and S7. In the part of the research carried out on BW25113 (Figure 2A), the fluorescence intensity was slightly, but repetitively, more intensive in cells treated with S2 and S7 compared to Ag^+^ treated cells. In contrast to the wt strain, the level of ROS generated within the mutant’s cells (AgR) depends on the used silver compound and incubation time (Figure 2C). An increase of the fluorescence intensity was observed in the case of samples treated with Ag^+^ (after 60 min) and S7 (after 30 min), but both signals were weaker than those derived from positive controls (with H_2_O_2_ and menadione). Measurement of the sample incubated with S2 showed a slight shift of fluorescence intensity comparable to the signal given by the control with the same bacteria. Probably S2 silver nanoformulation changes the cell wall structure, causing less permeability than the wild type of this strain. An unusual situation was observed during the experiment carried out on *Escherichia coli* J53. Even after a prolonged time of incubation of selected fluorescent dyes (DCFH-DA or DHE) with bacteria alone, some groups of bacterial cells remained unstained. That caused some problems, because it was difficult for the flow cytometer to detect a sufficient amount of bacterial cells for the reliable measurement. However, this problem affected only the strain *E. coli* J53 (Figure 2E). Histograms of the samples incubated with silver compounds show a fluorescence signal from numerous, well-dyed cells. It seems that the exposure to these agents caused changes in the bacterial membrane permeability that allowed DCFH-DA (or DHE) to enter the cell. Among the tested compounds, only the S2 sample did not show an increase in fluorescence intensity within an hour. Bacteria incubated with Ag^+^ and S7 started to generate ROS immediately after 30 min, but later on, the fluorescence signal from the first sample became weaker. Level of ROS generated by bacterial cells exposed to S7 remains the same even after 60 min. *E. coli* J53 strain, remaining sensitive to S2 and S7 SNF when resistant to silver ions, produced a higher level of ROS after treatment with the S7 than the S2 sample. It may result from the difference in bioavailability of S2 and S7 silver, their interaction with cell structures or production of various undetected type of reactive oxygen species.

Usage of the second fluorescent dye (dihydroethidium, DHE) gives us further information about generation of reactive oxygen species within tested strains of *Escherichia coli*. This time, results of the measurement were quite different from those obtained with DCFH-DA. The most noticeable change concerns histograms of ROS level in *E. coli* BW25113 wt (Figure 2B). Samples treated with S2 and S7 did not show any shifts in fluorescence intensity, even after 60 min of incubation. The peak of the fluorescence signal for both samples resembles that given by the control with untreated bacteria. However, incubation of the bacterial cells with Ag^+^ resulted in an increase in the fluorescence intensity after 30 min. In sum SNF S2 and S7 generate a lower level of ROS in the wild type of *E. coli* BW25113 strain than Ag^+^. In the case of the *E. coli* AgR strain, with changes of cell structure (lacking OmpC, OmpF and CusS proteins), a higher level of ROS was detected in the cells treated with S7 and Ag^+^ than S2 (Figure 2D). Fluorescence intensity of the sample treated with Ag^+^ significantly increased just after 30 min. This time the level of ROS was higher than the levels detected in both positive controls. The histogram of the sample treated with S7 looks very unusual. Two visible peaks represent groups of bacterial cells with different fluorescence intensity. The fluorescence signals are getting stronger with incubation time, but only one of them exceeds the value of the control with the same bacteria. In the case of the sample after S2 treatment, shifts observed during measurement reached the same intensity as the control with menadione within 60 min. It may suggest that SNF S2 interacts in a different way with bacterial cells than Ag^+^ and S7.

As mentioned before, staining problems had an impact on the results of research carried out on *E. coli* J53. It can be seen from the example of controls. The rest of the samples were stained properly (Figure 2F). Increase of the fluorescence intensity was observed only in the case of samples treated with Ag^+^ and S7 (just after 30 min of incubation with these compounds). The situation resembles the one occurred with DCFH-DA, but intensity signals for both samples dyed with DHE are much stronger. The sample treated with S7 did not show any shifts and remained at the level of ROS generated by the control with untreated bacteria. Further explanation requires deeper studies in the field of uptake and efflux of the silver nanoformulation.

### Scanning Electron Microscopy Observation

**FIGURE 3:**
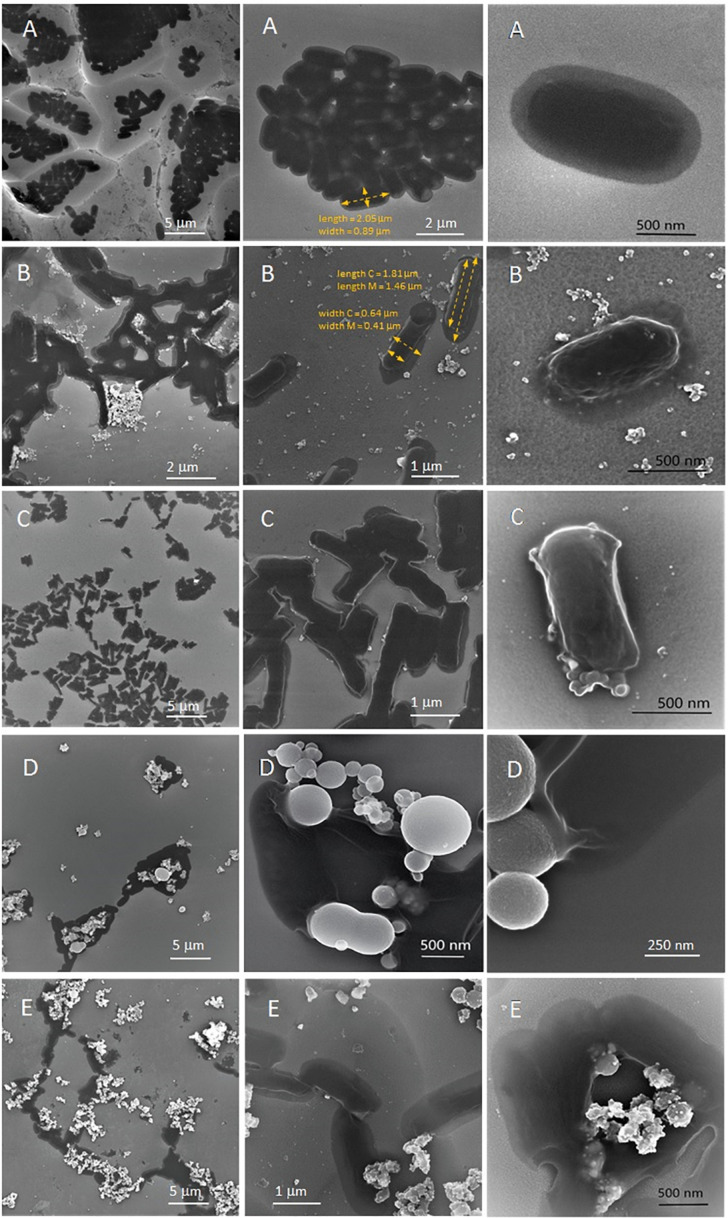
FE-SEM micrograps and quatified results on *E. coli* BW25113 bacteria before **(A)** and after treatment with silver ions or silver nanoformulation S2 or S7 **(B–E)**: Ag^+^ **(B)**, S7 **(C)**, pure TiO_2_ (silver carrier) **(D)** and S2 **(E)**. Legend: **(A)** Length = 2.10 ± 0.28 μm, Width = 0.81 ± 0.06 μm, oval shape, smooth surface; **(B)** Length C = 1.64 ± 0.19 μm, Width C = 0.61 ± 0.04 μm, Length M = 1.20 ± 0.16 μm, Width M = 0.43 ± 0.03 μm, distinctive structure with rough surface, flat area of the capsule, three-dimensional inner part surrounded by the cytoplasmic membrane area; **(C)** Length = 1.32 ± 0.15 μm, Width = 0.41 ± 0.04 μm, Length C = 1.38 ± 0.15 μm, Width C = 0.49 ± 0.05 μm, Length M = 1.29 ± 0.13 μm, Width M = 0.43 ± 0.05 μm, rectangular shape, distinctive structure with rough surface, in the case of some bacteria flat area of the capsule, three-dimensional inner part surrounded by the cytoplasmic membrane area; **(D)** Length = 1.74 ± 0.25 μm, Width = 0.78 ± 0.04 μm, oval shape, smooth surface; **(E)** Length C = 1.75 ± 0.26 μm, Width C = 0.80 ± 0.06 μm, Length M = 1.34 ± 0.24 μm, Width M = 0.45 ± 0.05 μm, distinctive structure with rough surface, flat area of the capsule, three-dimensional inner part surrounded by the cytoplasmic membrane area.

Using FE-SEM, we visualized the interaction of bacteria cells with silver ions and nanoforms (S2 and S7) and revealed changes in bacterial morphology (see Figure 3). Image Figure 3B (after Ag^+^ treatment) makes an impression as the disruption of bacterial membranes and leakage of cytoplasm. After SNF S7 exposure (Figure 3C) the deformation of cell structure was observed after nanoparticles embedded in the cell membrane. In the case of Figures 3D,E strong adhesion of nanomaterials (Figure 3D as pure TiO_2_ and Figure 3E for S2) to bacterial cells should be noted. In order to achieve a better dispersion of bacteria to measure their sizes, the samples were deposited on both the smooth and rough surfaces of the Si wafer. The length and width of bacteria were determined as the average size with a statistical deviation based on the measurement of several tens of individual bacteria. In the case of bacteria with smooth features, sizes were measured as highlighted in yellow in image Figure 3A. In the case of distinct structured shapes of bacteria, the external dimensions of the capsule (length C, width C) and the cytoplasmic membrane (length M, width M) were measured as highlighted in yellow in image Figure 3B. Detailed informations were described in figure legend (Figure 3).

### Circular Dichroism Measurement

**FIGURE 4:**
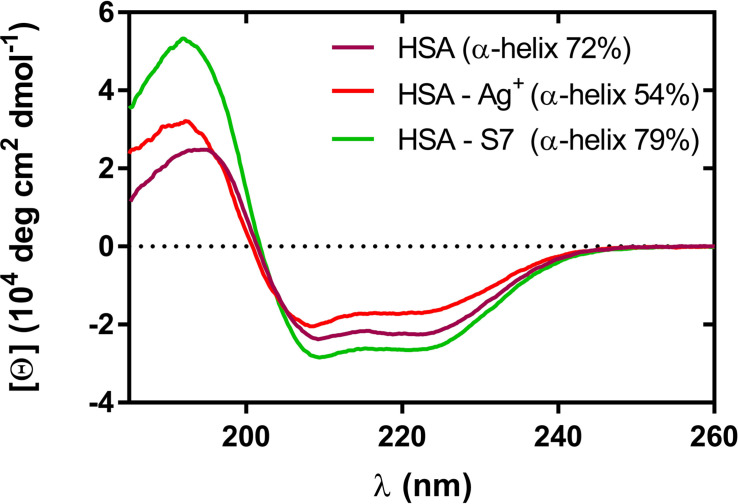
Circular dichroism spectra of HSA in the presence of AgNO_3_ and S7 sample. C(HSA) = 1.5 μM, C(Ag**^+^**) = 240 μM, C(S7) = 2 ppm. The fractional contents of HSA α-helices calculated from CD spectra by the Dichroweb platform (CDSSTR with dataset SP175) are described in the legend.

The differences in affinity to HSA between silver ions and nanoformulation S7 were identified via the CD assay. The circular dichroism results indicate that silver ions and nanoparticles (S7) cause conformational changes in HSA (Figure 4). The AgNO_3_ in a given concentration causes changes leading to a reduction in the amount of the HSA helical structure from 72 to 54% i.e., a denaturing effect. Sample S7 increases the amount of alpha helix, tending to stabilize the structure of HSA. This result is consistent with data obtained by Laera et al. (2011). The obtained results suggest differences in the mechanism of action between these forms of silver (ions and nanoparticles such as S7).

### Computational Studies

Due to the limitations of DFT calculations for such extended systems (here > 2500 heavy atoms), we present here a simplified model of the inner layer of ion channels where there is a sequence of interactions that are responsible for transport of Ag^0^ and Ag^+^ into the cell. The OmpC protein builds a three-way parallel hub of ion channels. We arbitrarily selected one of them (yellow in Supplementary Figure 1) in which an inner layer of 35 amino acids were chosen as available for interaction between metal and side chains.

To model the inner layer of the ion channel the following amino acids were taken into account: Tyr22, Asn26, Asp28, Gln33, Tyr35, Arg37, Glu43, Gln55, Gln59, Gln61, Asn63, Trp72, Arg74, Lys81, Gln83, Arg92, Tyr94, Tyr98, Asp105, Va216, Glu109, Tyr115, Ser117, Gln123, Arg124, Arg174, Lys226, Asp228, Tyr233, Gln264, Arg272, Tyr305, Lys308, Asn333 (see Supplementary Figure 2). Note that even for such an extensive range of amino acids, only a limited number of types of interaction between Ag and amino acid are possible, as only ten types of side chains are available for interaction with the metal.

All amino acids present inside of the ion channel can interact with Ag via their side chains. The interactions with Ag^0^ substantially differ from interactions with Ag^+^. Note that introduction of Ag^0^ to the channel changes multiplicity, but the total charge remains. Upon introduction of Ag^+^, the total charge changes, but multiplicity remains unchanged. To block unexpected interactions between the metal and C and N termini we replaced -Cα with the -CH_3_ group, noted as e.g., Asp-CH_3_, as presented in Supplementary Figure 3. In almost all investigated complexes distances between the side chains with Ag^0^ are longer, with Val-CH_3_ and Lys-CH_3_ being exceptions.

The investigated complexes can be divided into two groups. In the first group the pattern of interaction is similar for both Ag^0^ and Ag^+^, e.g., Arg-CH_3_, Asn-CH_3_, Gln-CH_3_, Lys-CH_3_, Ser-CH_3_, Val-CH_3_, Trp-CH_3_, and the main difference occurs only in metal-ligand distances.

In the second group differences between Ag^0^ and Ag^+^ lie not only in metal-ligand distances, but also in a different interaction pattern: Asp-CH_3_, Glu-CH_3_, Tyr-CH_3_. The side chain of Asp builds one or two interactions with the metal depending on the metal charge (see Supplementary Figure 3). In the complex with Glu-CH_3_ only one oxygen of the -COOH group is used, but positively charged Ag^+^ is pushed out from the carboxyl hydrogen area due to electrostatic repulsion. We observed a very interesting difference between complexes with Ag^0^ and Ag^+^ in complexes with Tyr-CH_3_: the Ag^+^ is connected via oxygen with the interaction pattern similar to that in the Ag^+^.Ser-CH_3_ complex, while Ag^0^ forms an interaction using an aromatic ring.

In the first group of complexes with similar structures of complexes with Ag^0^ and Ag^+^ the metal-ligand distances are in most cases significantly shorter (0.1–0.7 Å) for Ag^+^ complexes (see Supplementary Table 1).

Interestingly, the central area of the channel is only 10–11 Å in diameter, measured as the shortest distance between a heavy atom of side chains of the inner layer of the ion channel. At the same time the most remote interaction (Ag^0^.Tyr-CH_3_ complex) is about 3.4 Å. We can assume that the metal will interact only with one side of the channel at a time, upon passing through the channel.

The set of metal-ligand distances presented in Supplementary Table 1 describes all interactions inside the ion channel OmpC protein as rather weak. Using the supermolecular approach we calculated interaction energies (ΔE) for all complexes. As one would expect from the interaction distances, the energies differ substantially between Ag^0^ and Ag^+^ complexes. As presented in Supplementary Table 2, the series of Ag^0^.X-CH_3_ complexes form very weak interactions, in most cases a few kcal/mol, the values being similar to the energy of a single hydrogen bond. Such weak interactions allow Ag^0^ to very easily transfer from one complex to another upon descending into the cell.

The Ag^+^ complexes have greater interaction energies in comparison to Ag^0^ complexes. The difference between ΔE values can reach ∼20 kcal/mol (Asp-CH_3_). Two exceptions are complexes with Val-CH_3_ and Lys-CH_3_, where complexes with Ag^+^ are weaker than ones with Ag^0^, which are still very weak, with ΔE∼3.5 kcal/mol.

As passing through the OmpC ion channel has to engage consecutive series of complexes, passage via the channel for the Ag^+^ cation is more difficult due to the series of more effective interactions that have to be broken upon cation transfer (see Supplementary Table 2).

Similarly to the model of OmpC, we built a model on the inner layer of the ion channel for the OmpF protein (Supplementary Figure 4).

The inner layer of our model of the OmpF protein consists of the following residues: Lys10, Lys16, Leu20, Tyr22, Asn27, Glu29, Tyr32, Met38, Tyr40, Arg42, Lys46, Glu48, Gln60, Glu62, Gln66, Thr77, Lys80, Arg82, Leu83, Phe85, Lys89, Arg100, Tyr102, Tyr106, Asp113, Met114, Glu117, Asp121, Tyr124, Ser125, Arg132, Arg167, Arg168, Glu181, Lys219, Asp221, Asn224, Lys243, Asn246, Arg270, Tyr294, Tyr302, Lys305, Tyr310, Gln339 (see Supplementary Figure 5).

The set of interactions present in the ion channel of OmpF that shall be taken into consideration, beyond interactions described above, is Ag.X-CH_3_ where X = Leu, Met, Thr and Phe (Supplementary Figure 6).

Note that the metal-ligand distances for all four side chains are shorter for Ag^+^.X-CH_3_ by ∼0.2–0.5 Å (see Supplementary Table 3) in a range similar to that reported in Supplementary Table 1.

The analysis of the interaction energies leads to a similar pattern, as presented in Supplementary Table 2 – Met-CH_3_, Thr-CH_3_ and Phe-CH_3_ side chains build stronger interactions with Ag^+^, the exception here being Leu-CH_3_ (∼4 kcal/mol vs ∼1 kcal/mol), where the interaction energy is very similar to another aliphatic fragment – Val-CH_3_ (see Supplementary Table 4).

**FIGURE 5:**
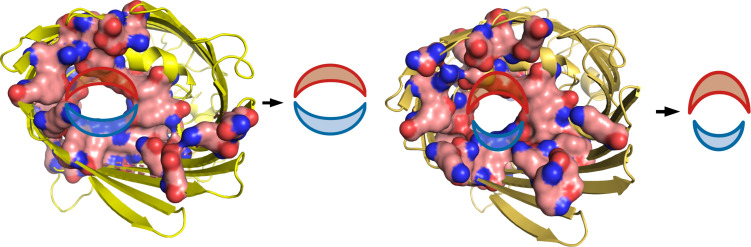
Oxygen- and nitrogen-rich areas in OmpC (left) and OmpF (right) ion channels.

Note that the Ag^+^ cation does not form a thermodynamically stable complex with Glu-CH_3_ (ΔE = + 51.6 kcal/mol), which implies that the paths inside the ion channel are different for Ag^0^ and Ag^+^ when the Glu side chain is present in the path of the metal descending. One can distinguish two gutter-like areas of the ion channel for both OmpC and OmpF proteins (Figure 5). The oxygen-rich path (Glu, Tyr, Ser, etc.) is indicated in red. The nitrogen-rich path, which consists mostly of Arg, is indicated in blue (see Supplementary Figures 2, 5). The nitrogen area is not energetically preferred by any form of Ag, and the Ag.Arg-CH_3_ complexes are the strongest for both Ag^0^ and Ag^+^ series. Interestingly, due to the energetically effective “oxygen path” being blocked by Glu, the Ag^+^ cations are forced to be transferred into the cell through the ion channel using at least some fragment of the less effective “nitrogen path.”

## Discussion

On the one hand, the use of nanosilver in antibacterial therapy as an alternative method of combating pathogens raises hopes due to its high biological activity, while on the other hand, it raises concerns related to the safety of use in the context of the development of bacterial resistance due to the fact that silver is ‘produced’ using various methods. Nanoparticles differ in a number of physico-chemical features (such as shape, size, surface development, chemical composition) from those known years ago. As we reported in our previous work (Kędziora et al., 2018) and other authors have reviewed (Pareek et al., 2018; Zheng et al., 2018), physicochemical properties (such as size, shape, compounds, oxidation state, crystallinity, charge) of silver nanoformulations qualify their interaction with bacterial cells, particles of biological importance and, finally, cell response (Kędziora et al., 2020). In this study we compared the mode of action between silver ions (Ag^+^) and silver nanoformulations (S2 – TiO_2_/Ag^0^ and S7 – aqueous dispersion of silver nanoparticles) and silver nanoformulations themselves (S2 and S7). The samples S2 and S7 differ from each other in size, compounds, surface area and bioavailability (Kędziora et al., 2020). Moreover, it should be added that environmental conditions (e.g., pH, chemical composition, temperature) determine the physico-chemical properties of nanomaterials and their behavior (Pareek et al., 2018; Siddiq et al., 2019). While the antibacterial mode of action (MoA) of silver ions is well defined and includes membrane disruption, ROS generation, molecules (such as protein, nucleic acids) inactivation, other studies confirm the various MoA after exposure to silver nanomaterials (Pareek et al., 2018; Bonilla-Gameros et al., 2020). In our previous work (Kędziora et al., 2020) we observed the different phenotypical (various silver and antibiotics’ sensitivity, silver resistance increase) and genetic changes (various types of mutations in structural and regulatory genes) in bacterial cells strongly depending on the kind of silver materials (silver ions, S2 and S7) which bacteria were exposed to. Zheng et al. (2018) reviewed the membrane disruption – pit formation, cell wall collapse and leakage – depending on the form of silver. As Anuj et al. (2019) observed, changed membrane permeability in a model *E. coli* strain was caused by cationic particles of nano-silver. They also suggested that membrane bound respiratory chain dehydrogenases obstruct the activities of efflux pump and DNA integrity. Finally, bacteria cells became more sensitive to linezolid than previously. Zheng et al. (2018) concluded that membrane damage could be related to ROS generation inside or outside the bacterial cell by silver nanomaterials. The visualization of the silver ions and SNF interactions made in our work draws attention to strictly specified interaction sites on the bacterial cell surface. On the one hand, the cell under the influence of S2 and S7 becomes susceptible to antibiotics, while on the other hand the level of detected ROS was very low (this work, Figure 2B). We observed a slightly higher ROS level after the Ag+ than in the S2 and S7 samples (Figure 2B). It should be emphasized that after treating *E. coli* BW25113 wt with S2 numerous point mutations in the wild type of *E. coli* BW25113 genome were noted while in the case of SNF S7 the number of point mutations was definitely lower (Kędziora et al., 2020). On this basis we may speculate that S2 and S7 have an additional mode of antibacterial action independent of ROS only, when the highest level of detected ROS was observed after exposure of bacteria to Ag^+^ (Figure 2B). These results are interesting to compare to computational methods where we demonstrated that investigated proteins (OmpC and OmpF) allow Ag^0^ to “slide” inside the cell more effectively – with a lower energy barrier in comparison to Ag^+^. The ROS investigation carried out by Borkowski et al. (2016) showed that *E. coli* cells treated with *SiC* (silicate carbon) nanofibers supported with Ag increased the concentration of reactive oxygen species within bacterial cells. The experiment was based on measurement of activity of catalase and dehydrogenase – enzymes involved in ROS detoxification. Similar results were obtained by Banerjee and his research team (Banerjee et al., 2010) using NBT (nitroblue tetrazolium) assay. Samples treated with Ag nanoparticles (NP) supported with chitosan and iodine did no show increased ROS generation in cells of *E. coli* (Banerjee et al., 2010). Differences in ROS level between samples with varied AgNP were an issue in the research of Kar et al. (2014) They observed an increase in ROS level after 1 h incubation of *E. coli* cells with AgNP and silver composites, but the concentration of free radicals was higher in the samples treated with AgNP. Zhang et al. (2018) used electron spin resonance spectroscopy (ESR) for detection of ROS in *Acinetobacter vinelandii* treated with silver nanoparticles differing in size (10 and 50 nm). The production of free radicals was higher in the samples treated with smaller nanoparticles and peaked after 4 h of incubation. They observed that the level of ROS generated in bacterial cells is dose-dependent. Similar results were obtained by Wu et al. (2019) when they used DCFH-DA to detect ROS in the cells of *Pseudomonas stutzeri* after exposure to AgNP with size of 15 nm. It may explain the difference in ROS generation between silver ions and tested SNF, which possess various bioavailability for the cell and ability to penetrate into the cell (Ag^+^ > S7 > S2).

An important aspect in the discussion on the mode of anti-microbial activity of SNF is the construction of the bacterial cell envelope. Li et al. (1997) reported that that mutations in porin-coding genes lead to changes in the sensitivity of *E. coli* to silver and that porins are involved in Ag^+^ uptake. Radzig et al. (2013) investigated the effect of mutations encoding porins OmpF and OmpC leading to changes in *E. coli* sensitivity to silver and silver nitrate nanoparticles. In the case of silver nitrate mutants lacking the OmpF porin and/or OmpC were more resistant to silver ions in relation to the wild strain. MIC values inhibiting bacterial growth for cells lacking some OMP indicate their greater resistance to silver nanoparticles compared with wild-type strain cells (they show a 4-8-fold increase in the resistance to silver nanoparticles than silver ions) (Li et al., 1997). Moreover, Liu et al. (2012) confirmed also that loss of the outer membrane protein C in *E. coli* is associated with the resistance to some antibiotics such as carbapenems and cefepime. Paradoxically, *E. coli* BW25113 mutant (AgR), although devoid of OmpC, OmpF, OmpR and CusS, and *E. coli* J53, resistant to silver ions, showed a higher level of detected ROS after treatment with Ag^+^ and S7 (Figure 2D) than the susceptible wild-type strain (no differences were observed when treated with S2).

The environmental conditions may be a separate cause of the interaction of silver nanoformulations with proteins. Siddiq et al. (2019) investigated the effect of the pH of the medium and behavior of silver nanoparticles with bovine serum albumine (BSA) using the CD technique. They concluded that α-helix decreased by increasing the AgNP concentration, and the basic medium fosters aggregation of silver nanoparticles. Our studies showed that at pH 8.3 α-helix decreases in the case of silver ions and increases in the case of silver nanoformulation S7. The results obtained by Siddiq et al. (2019) may suggest that more silver ions are related to HSA than silver nanoformulations (S7) (pI HSA and BSA are the same, equal to 4.7). Reymond-Laruinaz et al. (2016) in their studies confirmed the interaction of silver nanoparticles with proteins (including BSA). They found that silver nanoparticles are surrounded by a protein layer about 3-15 nm thick. It is also interesting that Galazzi et al. (2019) found differences in protein mass and activity of some enzymes (including those related to oxidation stress) in soybean seeds after treating them with silver ions (AgNO_3_) and silver nanoparticles (with average size 60 nm and spherical morphology) in the same concentration.

## Conclusion

We observed differences in the mode of antibacterial action between silver ions (Ag^+^) and silver nanomaterials (SNF, S2 and S7) and SNF (S2 and S7) themselves. Our results suggest that multiple OMP proteins are responsible for uptake of silver ions and silver nanoformulations. SNF were more efficacious against all tested bacterial strains than silver ions, and this was confirmed with computational methods: weaker interactions of Ag^0^ with amino acids of inner layers of both investigated proteins allow Ag^0^ to “slide” inside the cell more effectively – with a lower energy barrier in comparison to Ag^+^. Silver ion resistant strains (*E. coli* J53 and *E. coli* BW25113ompRG596AcusSG1130A) remained highly susceptible to tested silver SNF. This may result from a different mode of action of Ag^+^ and individual SNF. The level of ROS detected in bacterial cells depends on the phenotype of the bacterial strains. Strains resistant to Ag^+^ and SNF produce lower levels of ROS than silver sensitive ones, but in general Ag^+^ ions generate ROS more efficiently than SNF (S2 and S7). We noted that SNF were embedded in the cell structure and there was strong adhesion of SNF S2 to bacterial cells as observed by FE-SEM. Albumin structure was stabilized with SNF S7, while it was destabilized with silver ions. Fourteen types of amino acids side chains compose the inner layer of ion channels of OmpF and OmpC proteins. Both Ag^0^ and Ag^+^ forms display moderate interactions with side chains of amino acids inside the ion channels of OmpF and OmpC proteins. Two areas of the inner layer of the ion channel were observed: one, more effective, with oxygen-rich side chains; and another one, less effective, with nitrogen-rich side chains. The Glu side chain forces the Ag^+^ ion to use at least a fragment of the less effective nitrogen path of the ion channel.

## Data Availability Statement

The original contributions presented in the study are included in the article/Supplementary Material, further inquiries can be directed to the corresponding author/s.

## Author Contributions

AK: conceptualization. AK, RW, MS, IM, IL, and TG: methodology and data analysis. AK, RW, MS, IL, and TG: writing – original draft preparation. RW, GB-P, and VM: writing – review and editing. All authors contributed to the article and approved the submitted version.

## Conflict of Interest

The authors declare that the research was conducted in the absence of any commercial or financial relationships that could be construed as a potential conflict of interest.
