# Supplementary material for: Comparison of Antibacterial Mode of Action of Silver Ions and Silver Nanoformulations With Different Physico-Chemical Properties: Experimental and Computational Studies
Source: Front Microbiol. 2021 Jul 1;12:659614. doi: 10.3389/fmicb.2021.659614 (PMC8281304; doi:10.3389/fmicb.2021.659614)
Supplement: Supplementary file 1 [file Data_Sheet_2.docx]

Supplementary Material

Supplementary note: Figures 1–6 and Tables 1-4.

**Table of contents:**

**Supplementary Figure 1:** The model of OmpC protein used for DFT calculations.

**Supplementary Figure 2:** Side chains of inner layer of the OmpC protein ion channel.

**Supplementary Figure 3:** The X-CH_3_ complexes with Ag where X=Arg, Asn, Gln, Lys, Ser, Val, Trp, Asp, Glu, Tyr.

**Supplementary Figure 4:** The model of OmpF protein used for DFT calculations.

**Supplementary Figure 5:** Side chains of inner layer of the OmpF protein ion channel.

**Supplementary Figure 6:** The X-CH_3_ complexes with Ag where X=Leu, Met, Thr and Phe.

**Supplementary Table 1:** Metal – ligand distance in Å. OmpC protein.

**Supplementary Table 2:** Metal – ligand energies of interactions in OmpC protein.

**Supplementary Table 3:** Metal – ligand distances in Å, for Ag..X-CH_3_ where X=Leu, Met, Thr and Phe.

**Supplementary Table 4:** Metal – ligand energies for Ag..X-CH_3_ where X=Leu, Met, Thr and Phe (OmpF protein).


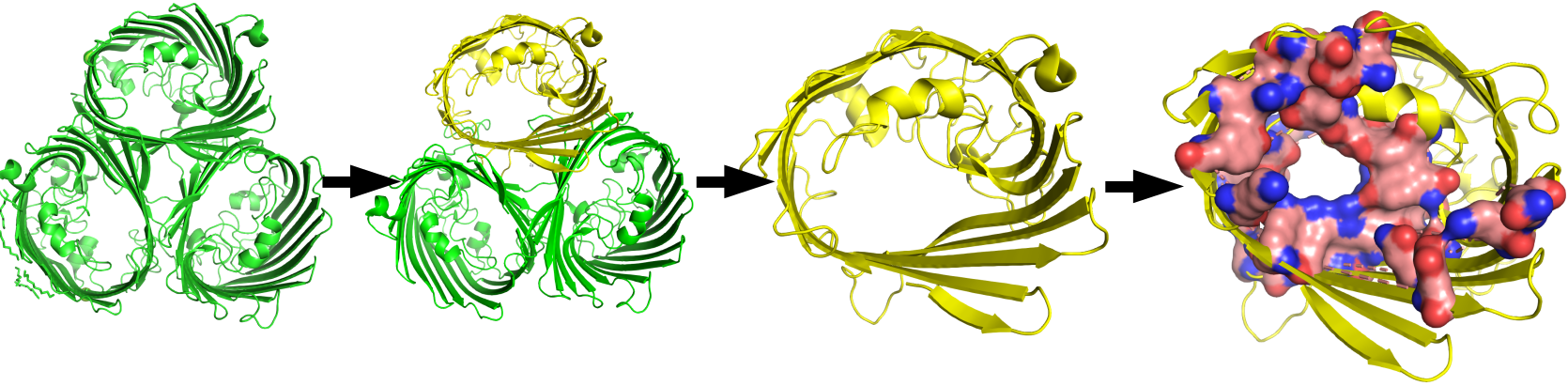


**Supplementary Figure 1:** The model of OmpC protein used for DFT calculations.
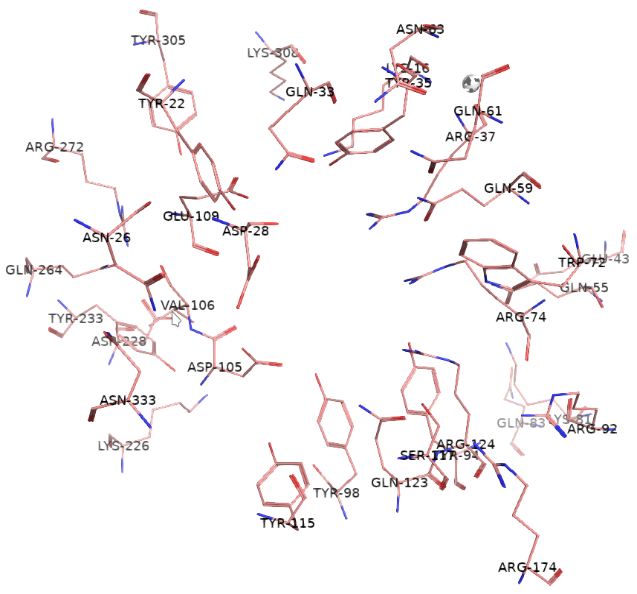


**Supplementary Figure 2:** Side chains of inner layer of the OmpC protein ion channel.


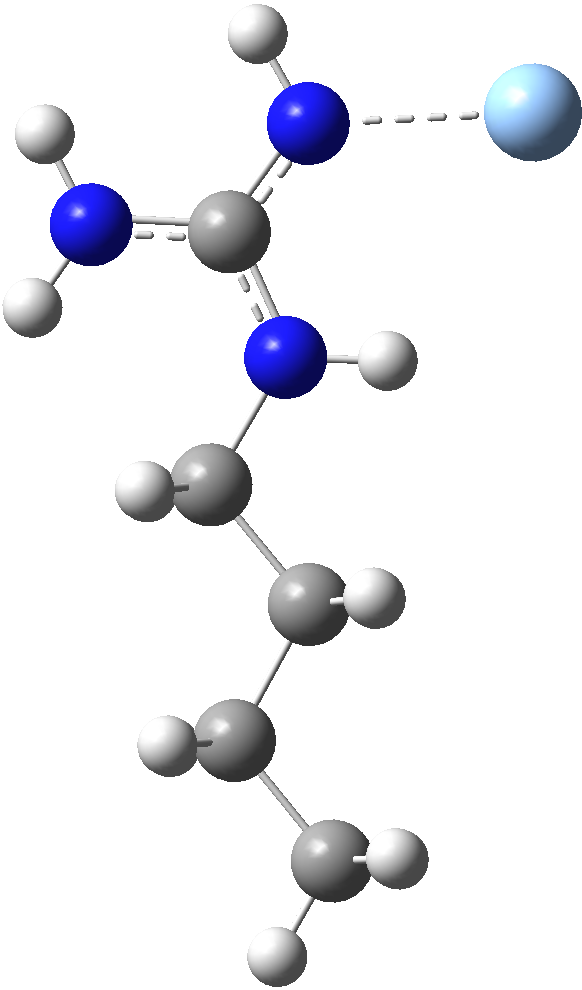


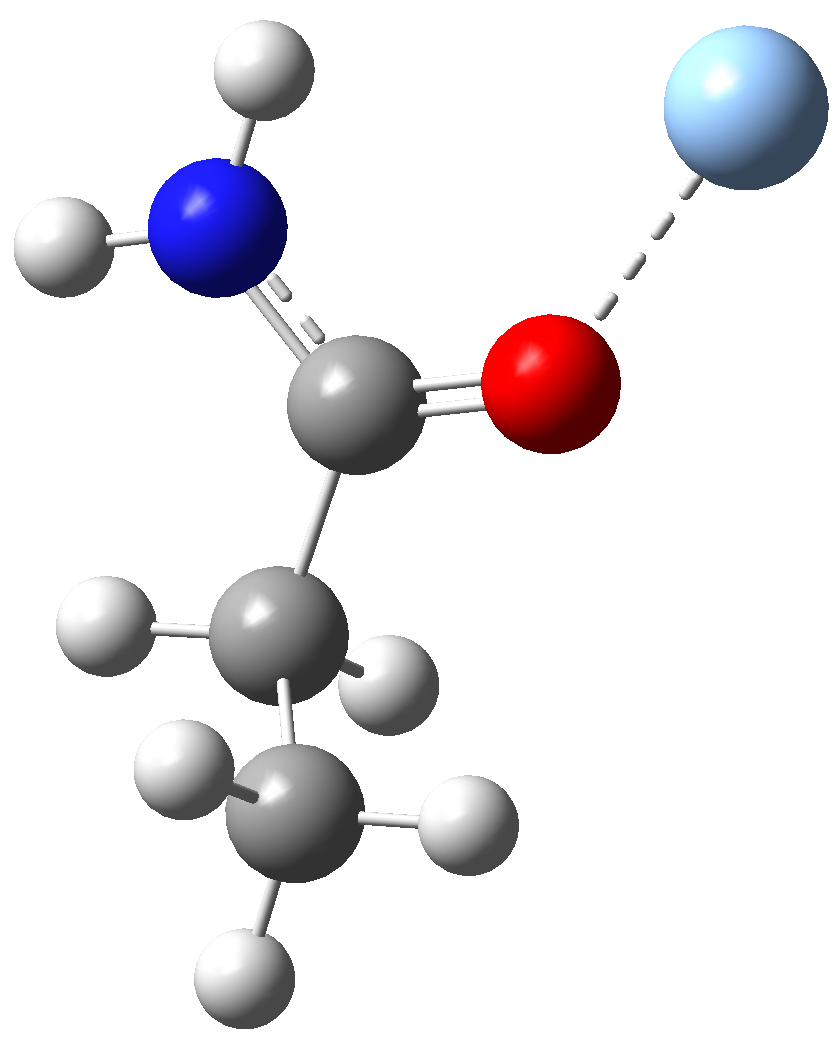

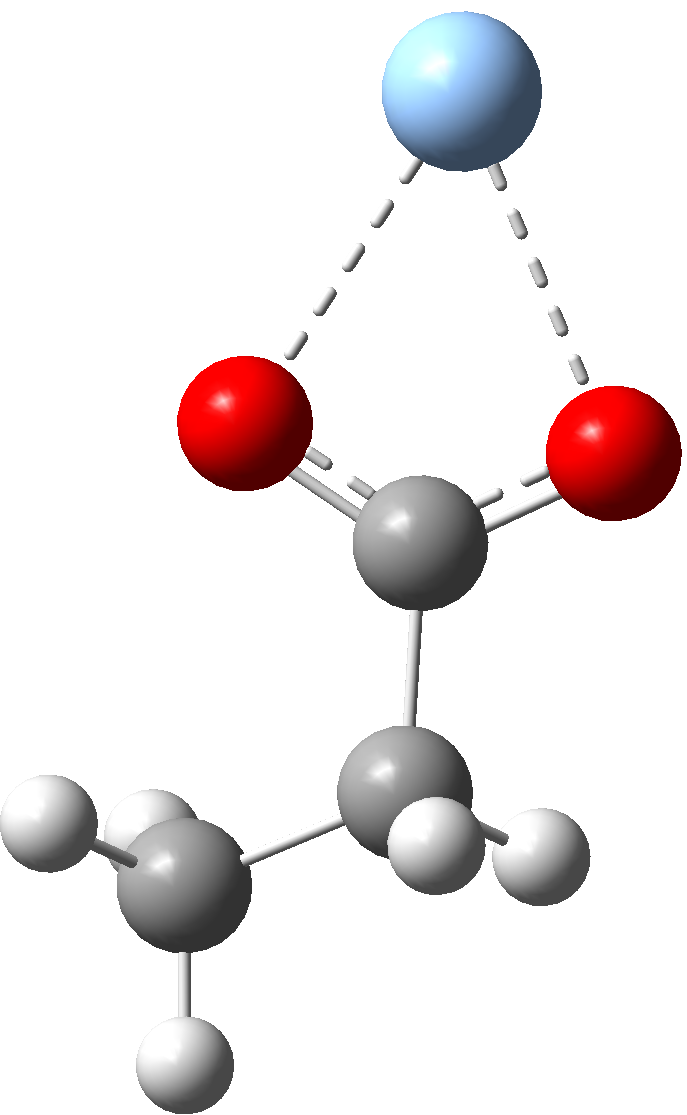

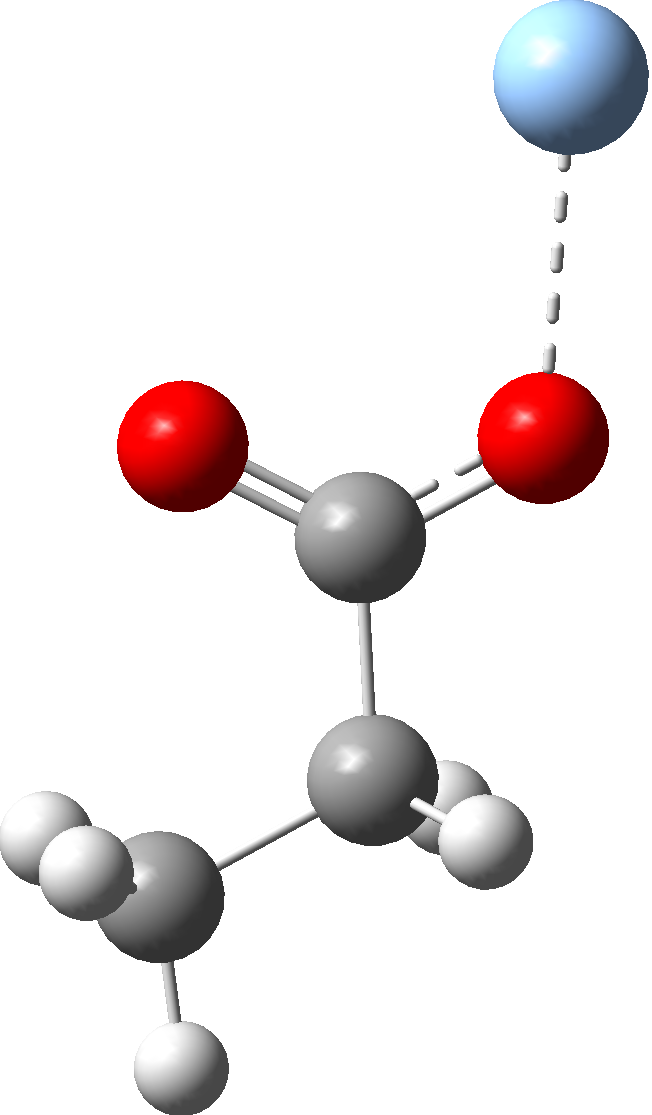

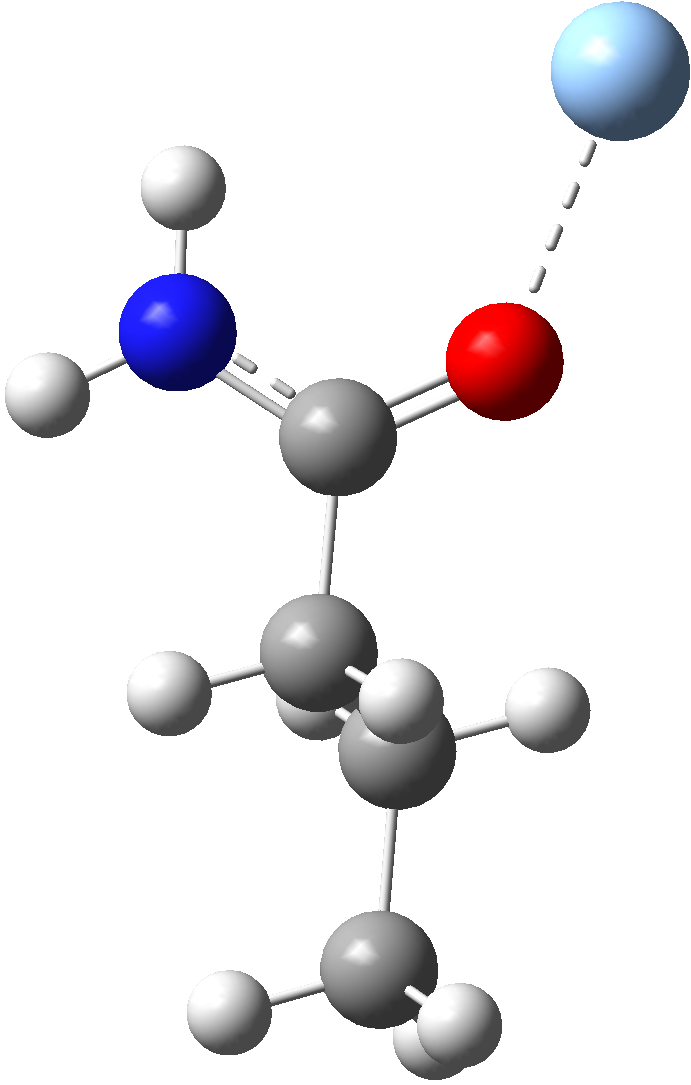


Ag..Arg-CH_3_ Ag..Asn-CH_3_ Ag^+^..Asp-CH_3_ Ag^0^..Asp-CH_3_ Ag..Gln-CH_3_


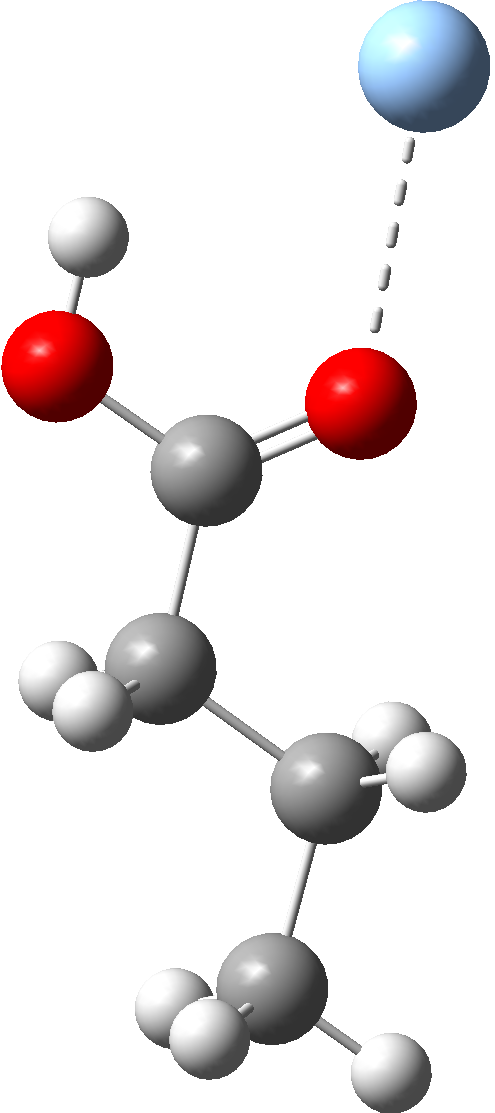

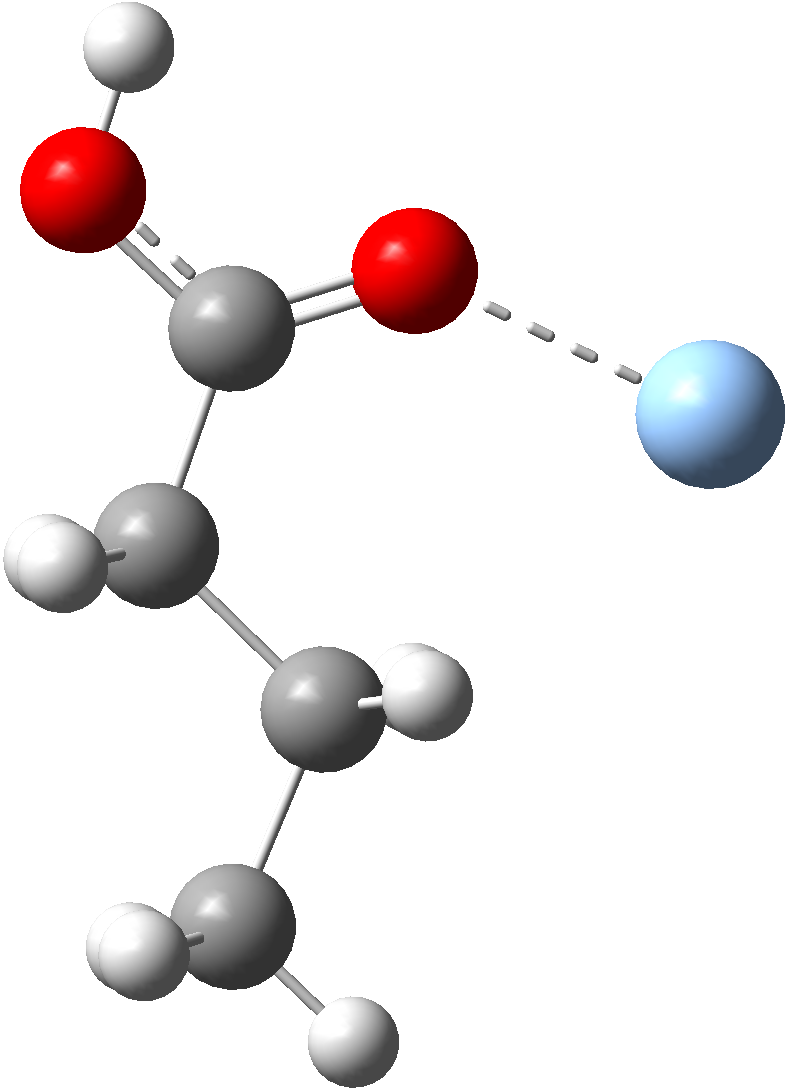

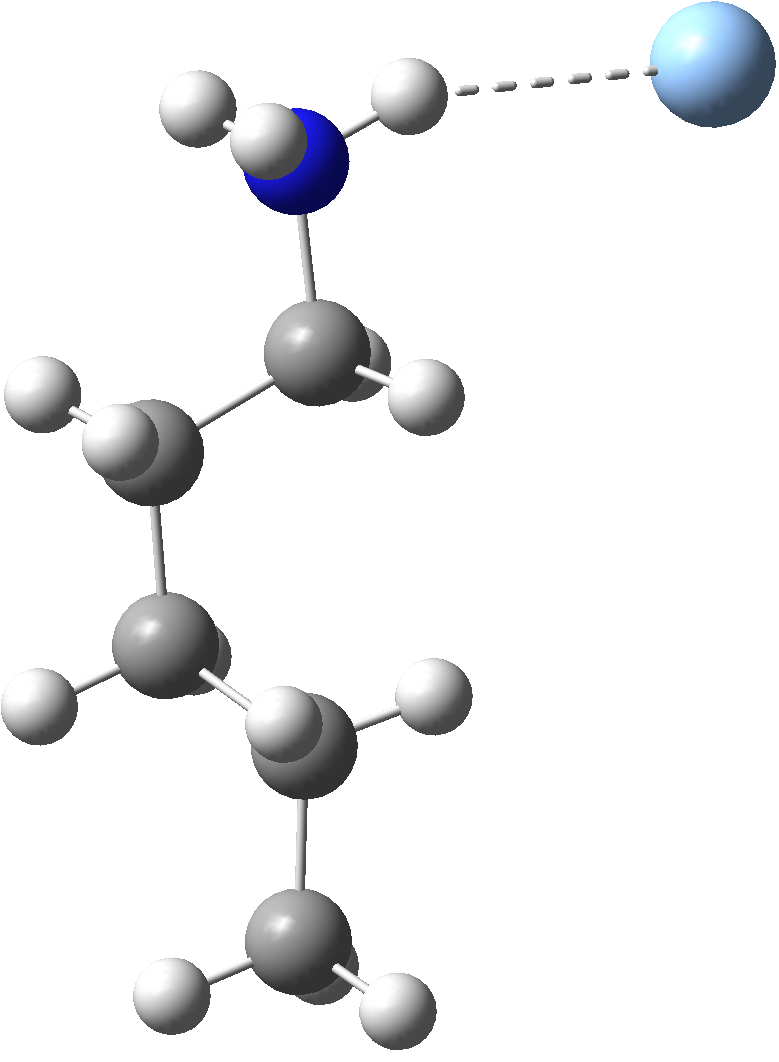

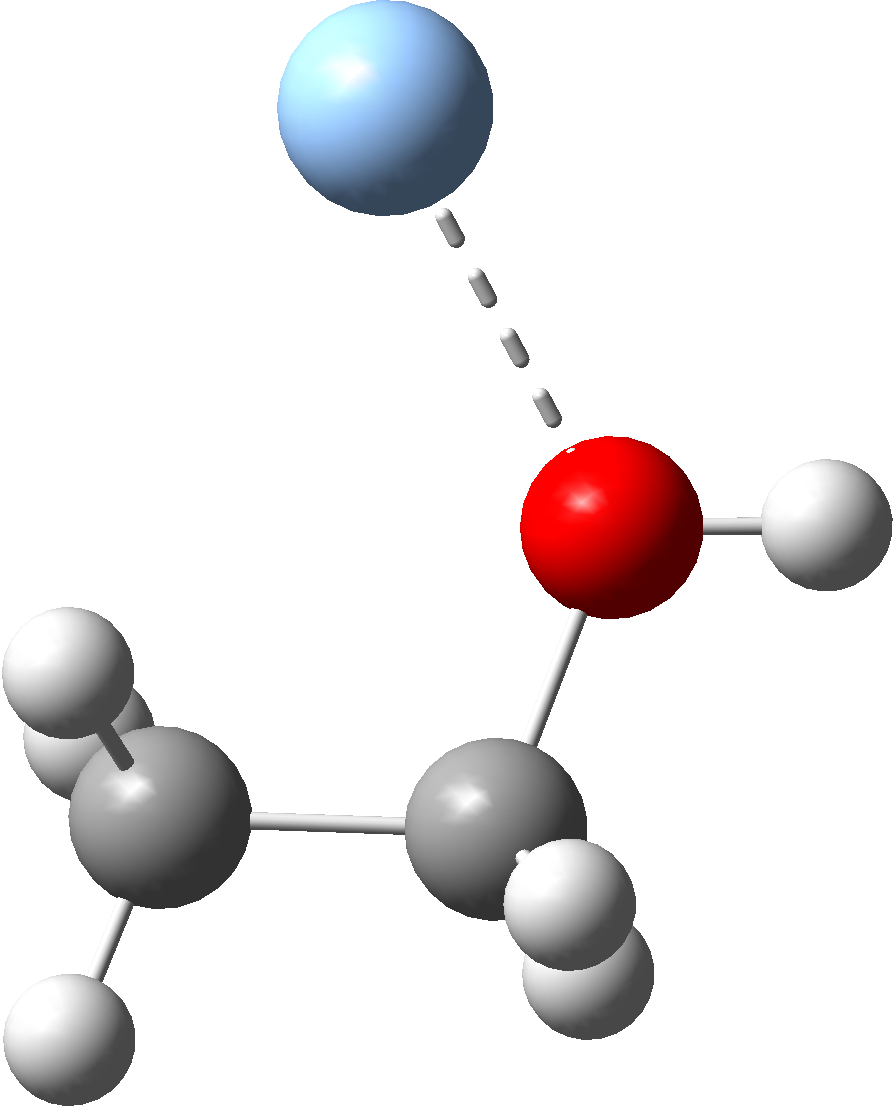

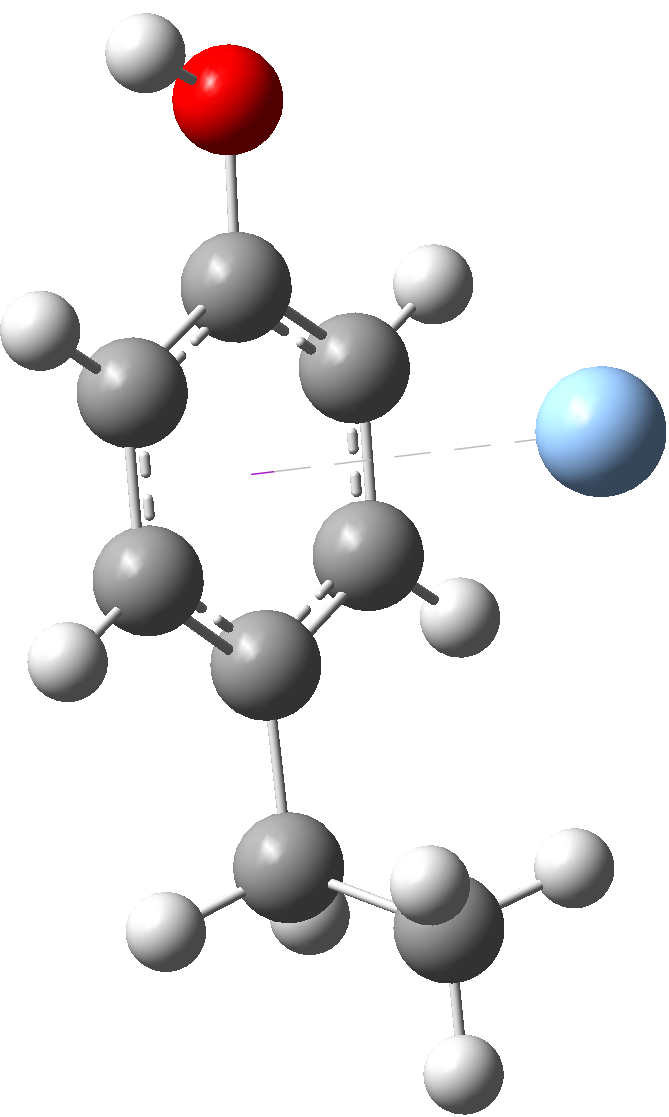


Ag^0^..Glu-CH_3_ Ag^+^..Glu-CH_3_ Ag..Lys-CH_3_ Ag..Ser-CH_3_ Ag^0^..Tyr-CH_3_


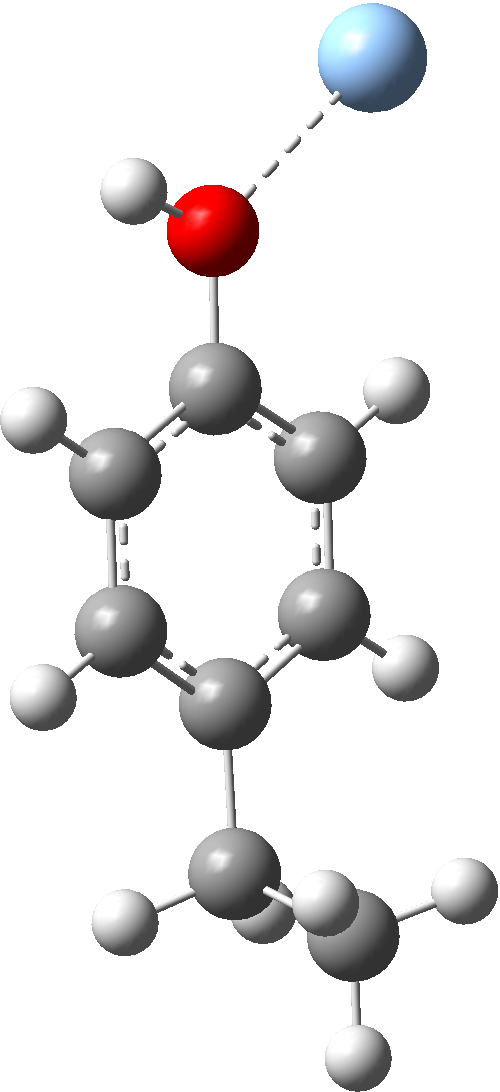

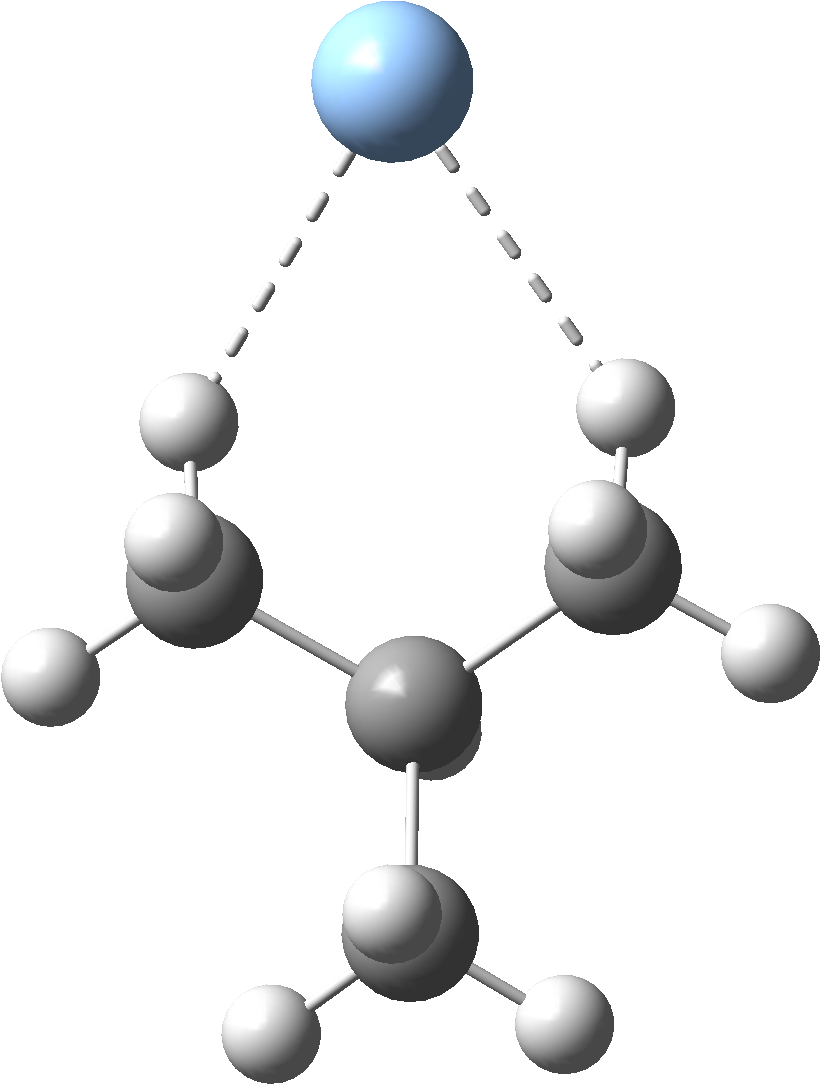

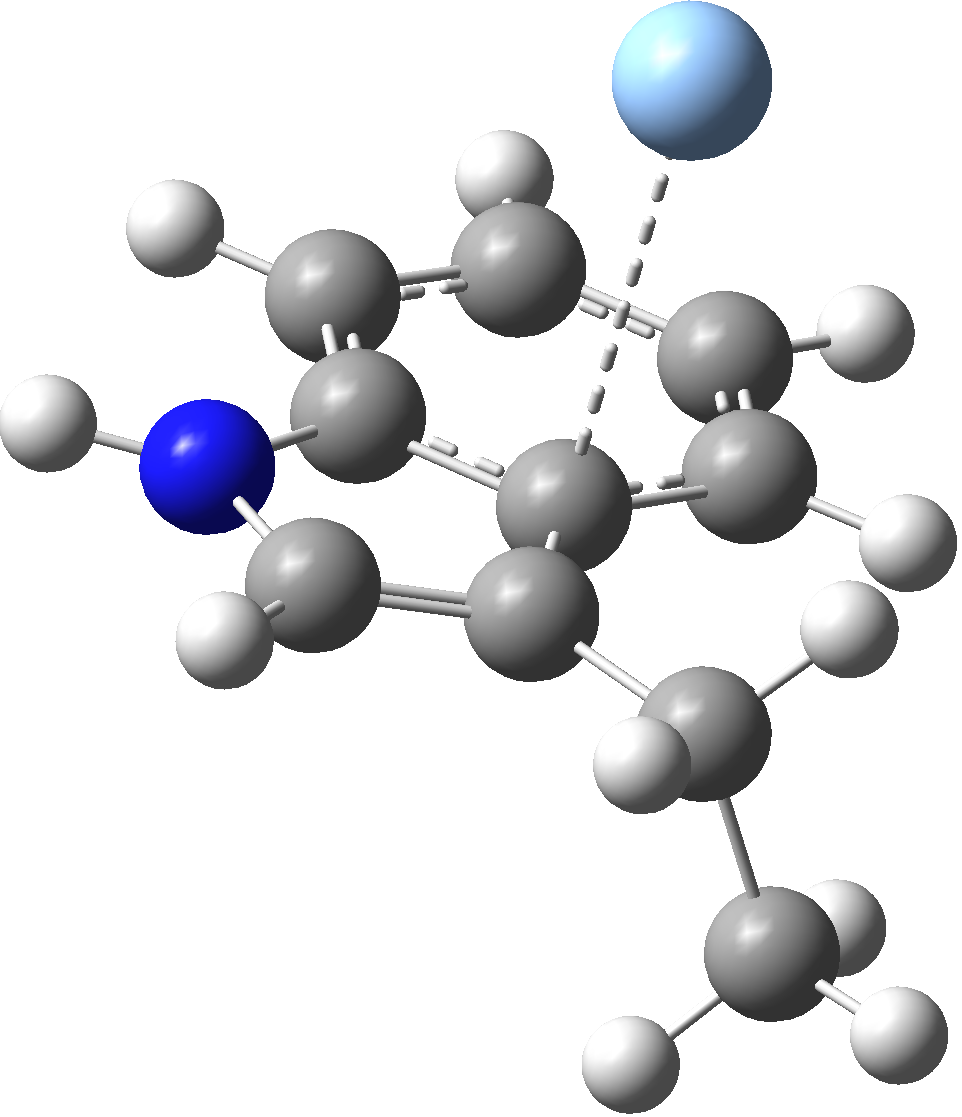


Ag^+^..Tyr-CH_3_ Ag..Val-CH_3_ Ag..Trp-CH_3_

**Supplementary Figure 3:** The X-CH_3_ complexes with Ag where X=Arg, Asn, Gln, Lys, Ser, Val, Trp, Asp, Glu, Tyr.


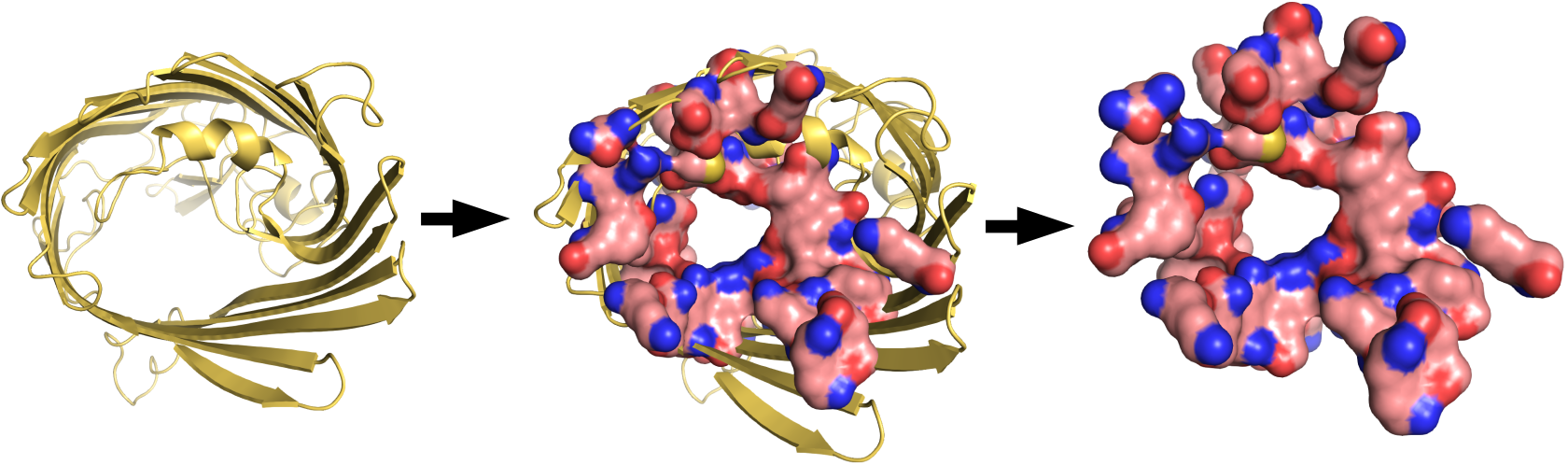


**Supplementary Figure 4.** The model of OmpF protein used for DFT calculations.


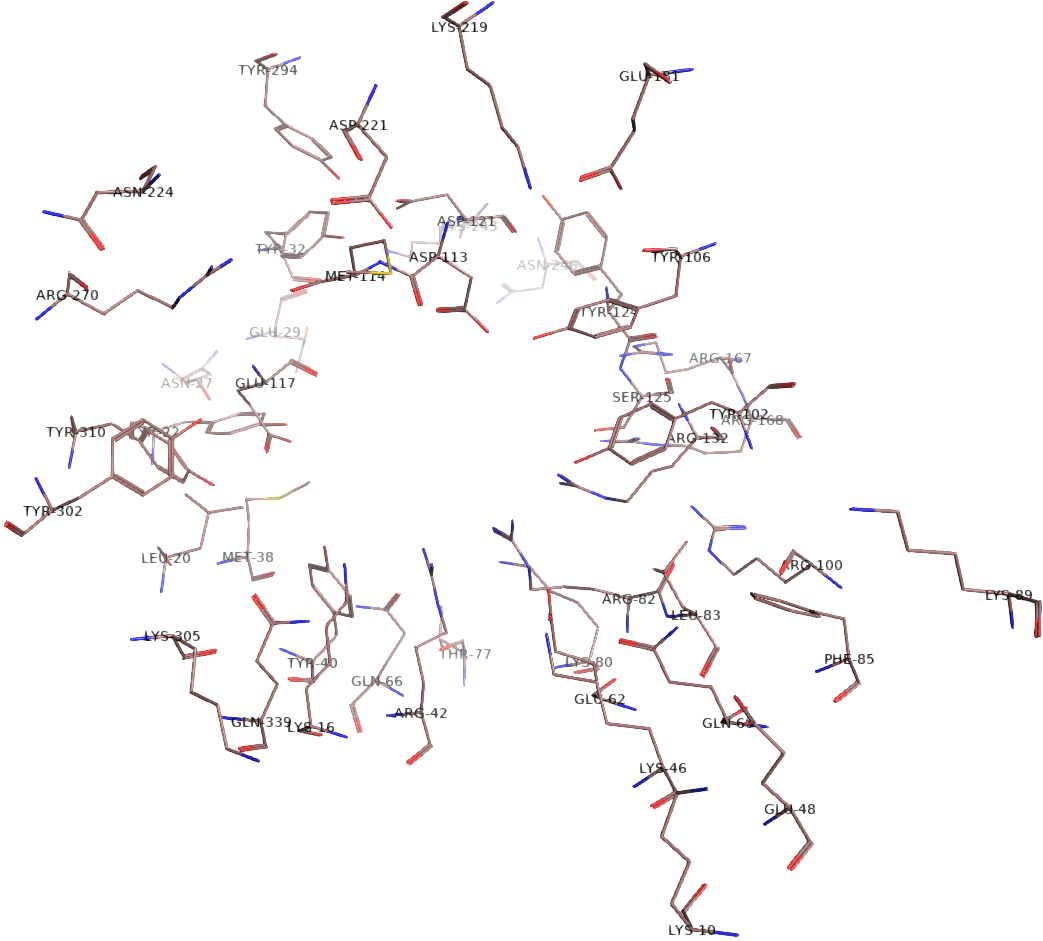


**Supplementary Figure 5:** Side chains of inner layer of the OmpF protein ion channel.


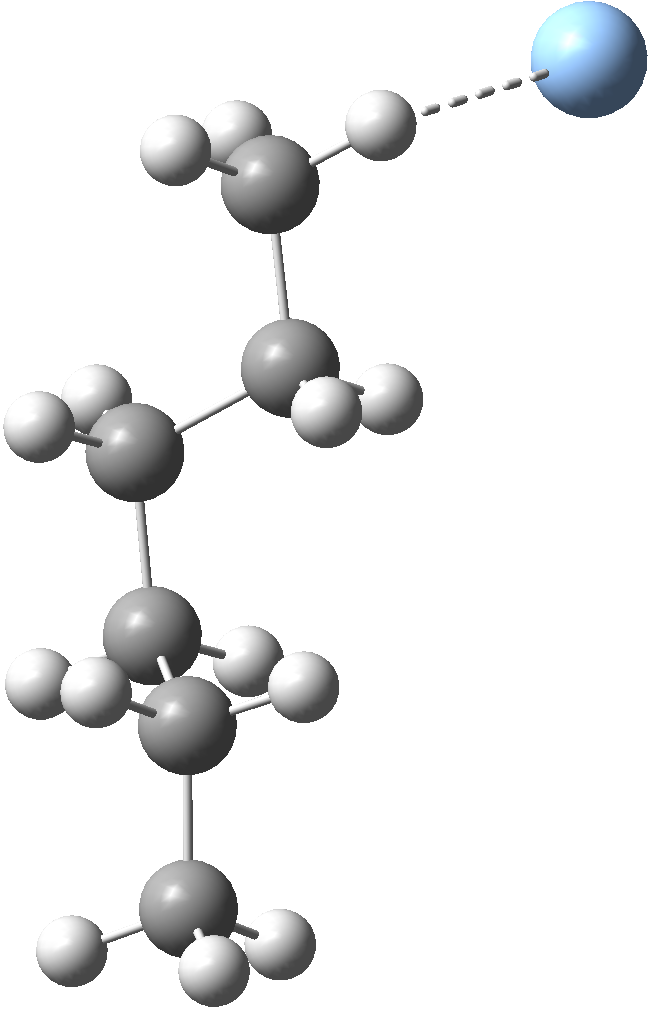

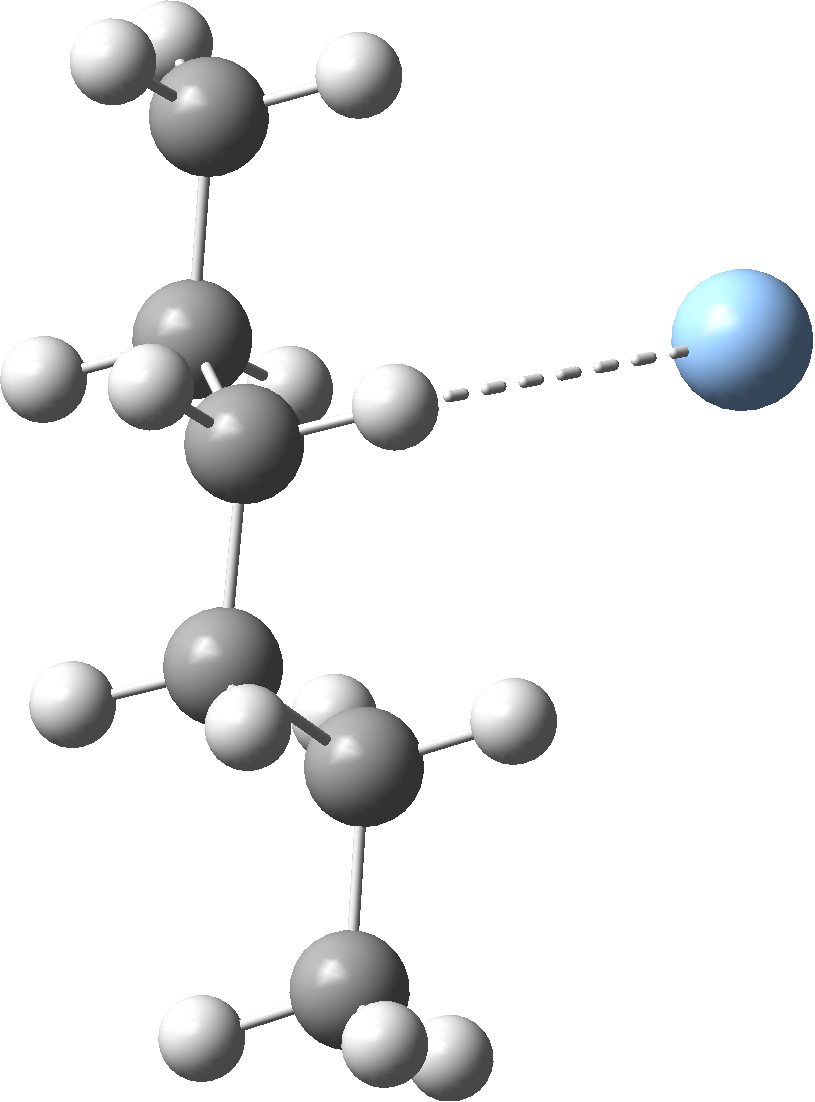

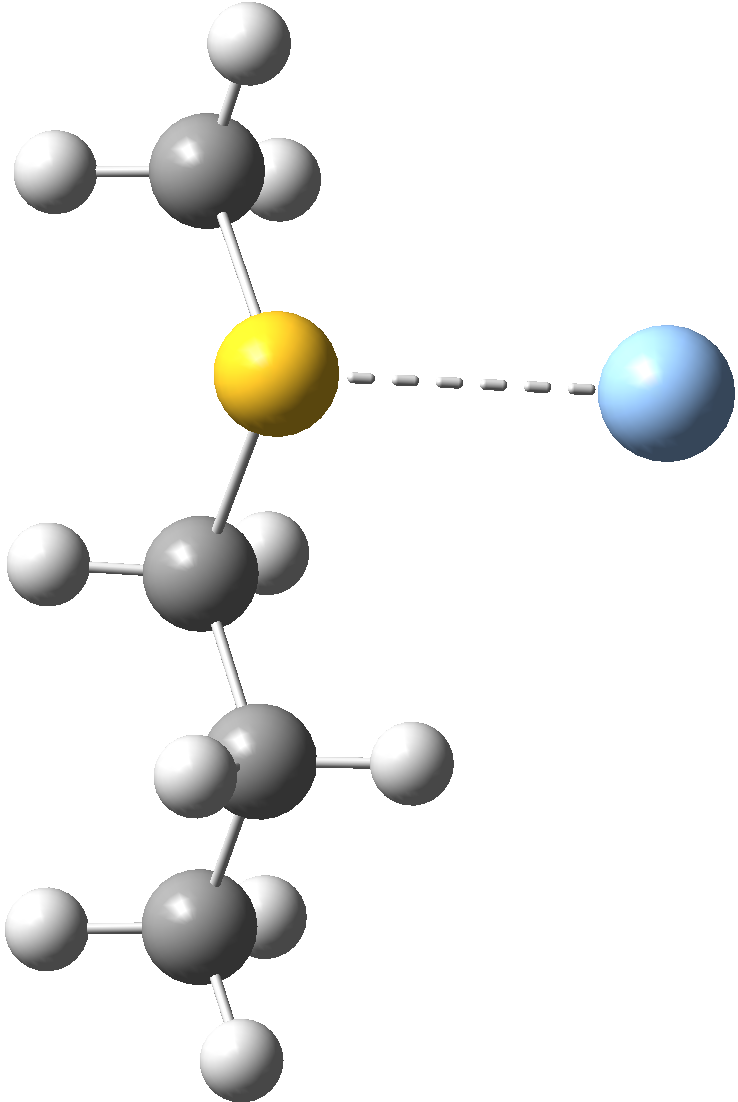

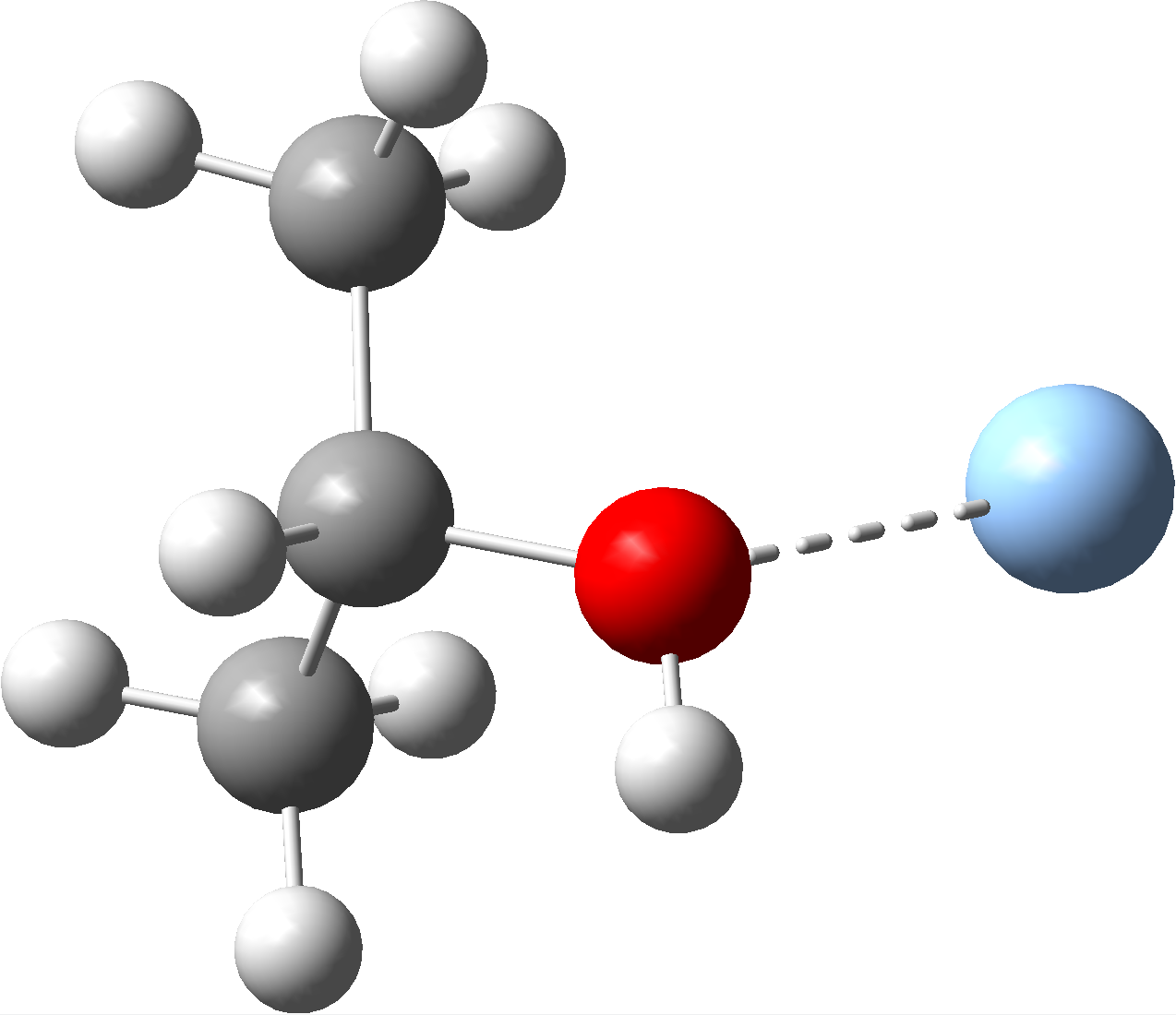

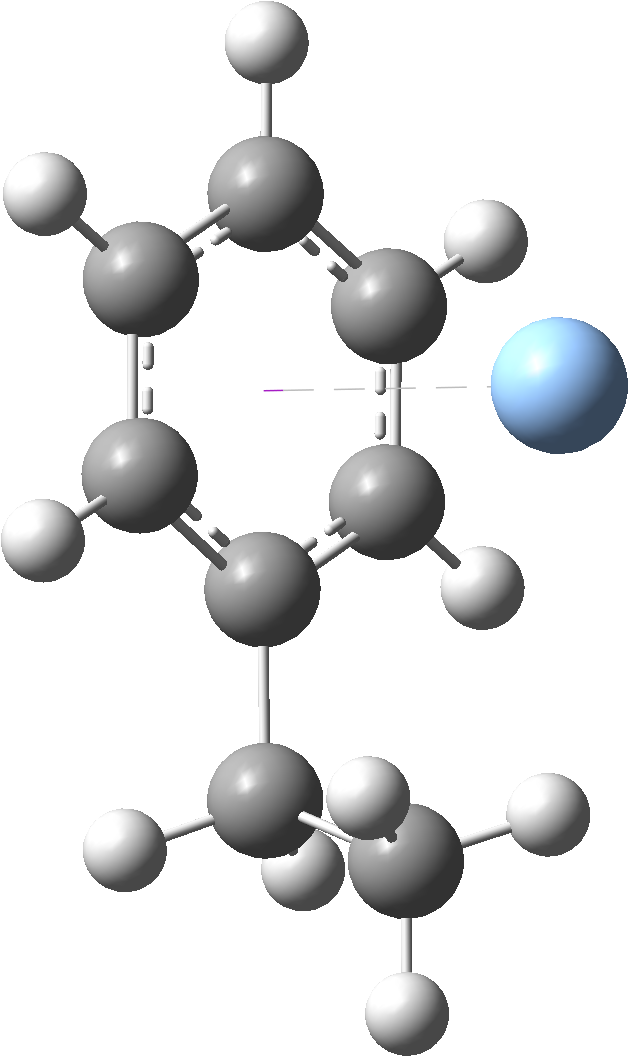


Ag^0^..Leu-CH_3_ Ag^+^..Leu-CH_3_ Ag..Met-CH_3_ Ag..Thr-CH_3_ Ag..Phe-CH_3_

**Supplementary Figure 6:** The X-CH_3_ complexes with Ag where X=Leu, Met, Thr and Phe.

| **X** | **Ag^0^..X-CH_3_** | **Ag^+^..X-CH_3_** | **Δ** |
| --- | --- | --- | --- |
| Arg | 2.230 | 2.129 | -0.101 |
| Asn | 2.436 | 2.201 | -0.235 |
| Asp (via O_1_) | 2.286 | 2.386 | 0.100 |
| Asp (via O_2_) | - | 2.415 | - |
| Gln | 2.429 | 2.206 | -0.223 |
| Glu | 2.544 | 2.168 | -0.376 |
| Lys | 2.579 | 3.305 | 0.726 |
| Ser | 2.446 | 2.240 | -0.206 |
| Tyr | 3.389 | 2.289 | -1.100 |
| Val | 2.421 | 3.059 | 0.638 |
| Trp | 3.217 | 2.489 | -0.728 |

**Supplementary Table 1:** Metal – ligand distance in Å. OmpC protein.

|  | X-CH_3_ | Ag^0^..X-CH_3_ | Ag^+^..X-CH_3_ | **Ag^0^..X-CH_3_** | **Ag^+^..X-CH_3_** |
| --- | --- | --- | --- | --- | --- |
|  | E [a.u.] | | | **ΔE [kcal/mol]** | |
| Arg | -362.4667 | -509.4637 | -509.3705 | **-13.8** | **-32.1** |
| Asn | -248.4334 | -395.4156 | -395.3117 | **-4.5** | **-16.1** |
| Asp | -267.8144 | -414.8063 | -414.7173 | **-10.6** | **-31.5** |
| Gln | -287.7305 | -434.7175 | -434.6089 | **-7.5** | **-16.2** |
| Glu | -307.57142 | -454.5533 | -454.3417 | **-4.3** | **51.6** |
| Lys | -253.4764 | -400.4570 | -400.3305 | **-3.5** | **-0.9** |
| Ser | -154.9797 | -301.9620 | -301.8614 | **-4.6** | **-18.3** |
| Tyr | -385.9291 | -532.9116 | -532.7997 | **-4.7** | **-11.3** |
| Val | -158.3884 | -305.3688 | -305.2439 | **-3.4** | **-1.8** |
| Trp | -442.2504 | -589.2327 | -589.1230 | **-4.6** | **-12.5** |

**Supplementary Table 2:** Metal – ligand energies of interactions in OmpC protein.

|  | Ag^0^..X-CH_3_ | Ag^+^..X-CH_3_ | Δ |
| --- | --- | --- | --- |
| Leu | 2.959 | 2.453 | -0.506 |
| Met | 2.832 | 2.549 | -0.283 |
| Thr | 2.473 | 2.230 | -0.243 |
| Phe | 3.664 | 3.456 | -0.208 |

**Supplementary Table 3:** Metal – ligand distances in Å, for Ag..X-CH_3_ where X=Leu, Met, Thr and Phe.

|  | X-CH_3_ | Ag^0^..X-CH_3_ | Ag^+^..X-CH_3_ | **Ag^0^..X-CH_3_** | **Ag^+^..X-CH_3_** |
| --- | --- | --- | --- | --- | --- |
|  | E [a.u.] | | | **ΔE [kcal/mol]** | |
| Leu | -236.9799 | -383.9615 | -383.8348 | **-4,1** | **-1,4** |
| Met | -556.5299 | -703.5195 | -703.4116 | **-9,1** | **-18,3** |
| Thr | -194.2828 | -341.2662 | -341.1611 | **-5,3** | **-16,1** |
| Phe | -310.7345 | -457.7223 | -457.6035 | **-8,0** | **-10,3** |

**Supplementary Table 4:** Metal – ligand energies for Ag..X-CH_3_ where X=Leu, Met, Thr and Phe (OmpF protein).
